# Production of Magnetic Arsenic–Phosphorus Alloy Nanoribbons with Small Band Gaps and High Hole Conductivities

**DOI:** 10.1021/jacs.3c03230

**Published:** 2023-08-08

**Authors:** Feng Fei Zhang, Eva Aw, Alexander G. Eaton, Rebecca R. C. Shutt, Juhwan Lim, Jung Ho Kim, Thomas J. Macdonald, Cesar III D. L. Reyes, Arjun Ashoka, Raj Pandya, Oliver D. Payton, Loren Picco, Caroline E. Knapp, Furio Corà, Akshay Rao, Christopher A. Howard, Adam J. Clancy

**Affiliations:** † Department of Chemistry, University College London, London WC1E 6BT, U.K.; ‡ Department of Physics and Astronomy, University College London, London WC1E 6BT, U.K.; § Cavendish Laboratory, Department of Physics University of Cambridge, Cambridge CB3 0HE, U.K.; ∥ Department of Materials Science and Metallurgy, University of Cambridge, Cambridge CB3 0FS, U.K.; ⊥ School of Engineering and Materials Science, Queen Mary University of London, London E1 4NS, U.K.; # Laboratoire Kastler Brossel, ENS-Université PSL, CNRS, Sorbonne Université, Collège de France, 24 rue Lhomond, 75005 Paris, France; g Interface Analysis Centre, H. H. Wills Physics Laboratory, University of Bristol, Bristol, BS8 1TL, U.K.

## Abstract

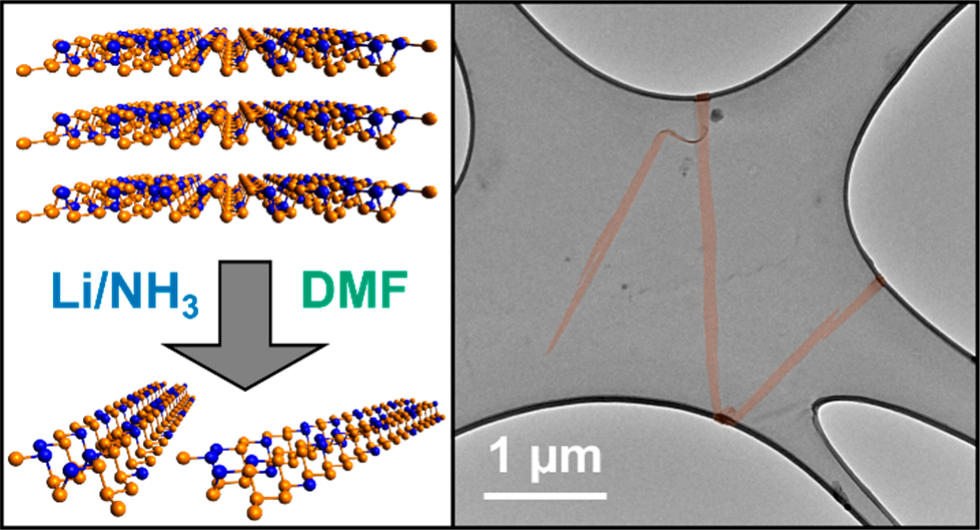

Quasi-1D nanoribbons provide a unique route to diversifying the properties of their parent 2D nanomaterial, introducing lateral quantum confinement and an abundance of edge sites. Here, a new family of nanomaterials is opened with the creation of arsenic–phosphorus alloy nanoribbons (AsPNRs). By ionically etching the layered crystal black arsenic–phosphorus using lithium electride followed by dissolution in amidic solvents, solutions of AsPNRs are formed. The ribbons are typically few-layered, several micrometers long with widths tens of nanometers across, and both highly flexible and crystalline. The AsPNRs are highly electrically conducting above 130 K due to their small band gap (ca. 0.035 eV), paramagnetic in nature, and have high hole mobilities, as measured with the first generation of AsP devices, directly highlighting their properties and utility in electronic devices such as near-infrared detectors, quantum computing, and charge carrier layers in solar cells.

## Introduction

Phosphorene, a corrugated 2D sheet of phosphorus, has been the subject of intense study owing to its high carrier mobility, layer-dependent direct band gap, high surface area, and theoretical energy density. Phosphorene is typically produced by exfoliation from its parent layered crystal, black phosphorus (bP), itself often synthesized either through crystallization from a heavy metal-melt or through chemical vapor deposition of phosphorus precursors. To integrate phosphorene into contemporary devices, key issues must be addressed including scaling its synthesis,^1^ improving its stability in air,^2^ and directing its properties to better suit a desired practical application/device. The properties of phosphorene may be modified through a range of approaches including functionalization,^3^ doping,^4^ and strain.^5^

**Figure 1:**
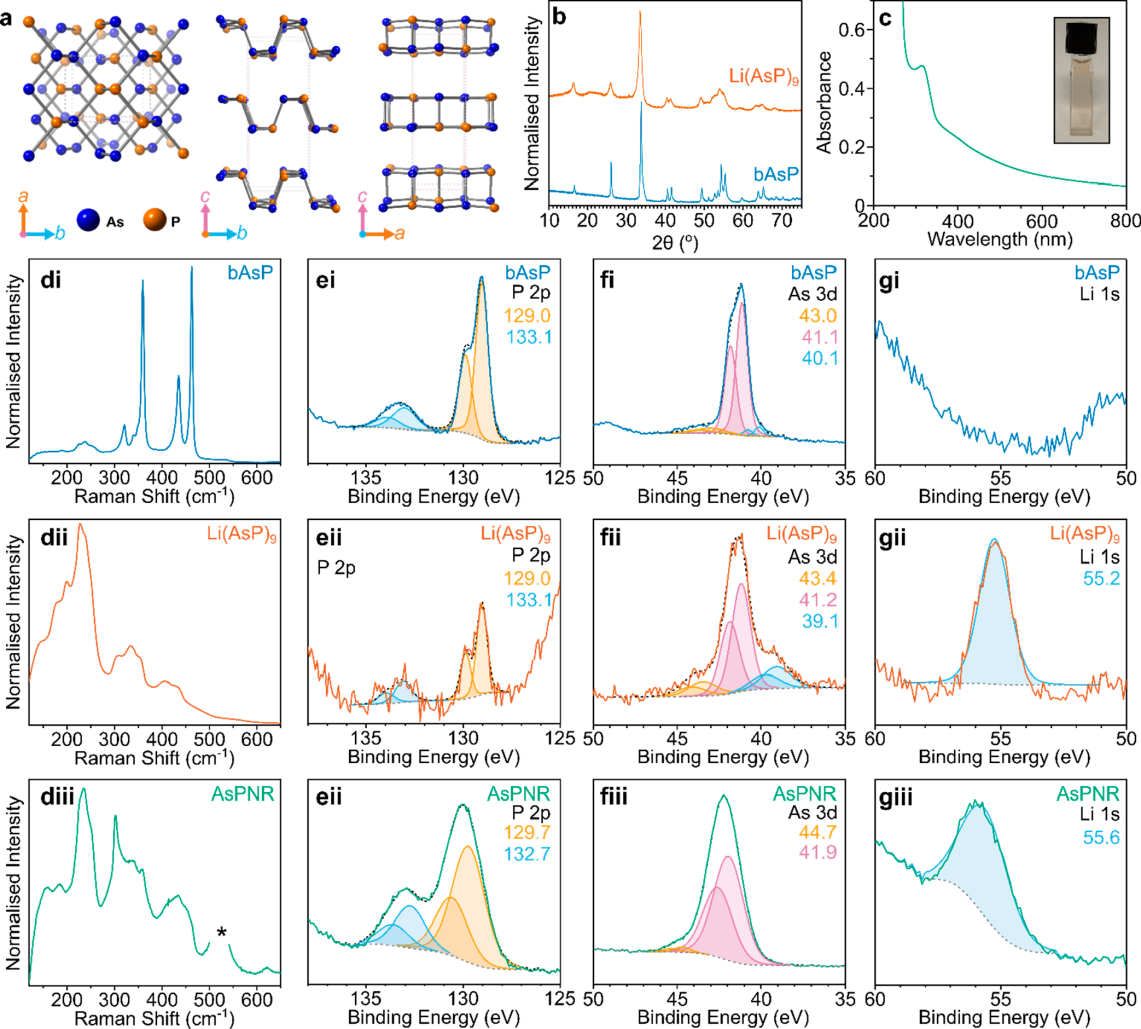
(a) Structure of bAsP crystal highlighting random arrangement of As/P atoms. Structure is shared for AsPNR basal plane. (b) pXRD of bAsP and Li(AsP)_9_, larger reproduction provided in SI, Figure S2. (c) UV–vis spectrum of AsPNR solution in DMF, picture of cuvette inset. (d) Raman spectra averaged over 121 normalized spectra at points separated by ≥5 μm of (i) bAsP, (ii) Li(AsP)_9_, and (iii) AsPNRs drop-cast onto silicon from DMF solution; * 500–540 cm^–1^ region removed due to intense silicon peak. Additional Raman spectra are provided in SI, Figure S4. (e–g) XPS spectra of (i) bAsP, (ii) Li(AsP)_9_, and (iii) AsPNRs drop-cast from DMF solution in (d) P 2p, (e) As 3d, and (f) Li 1s regions with peak centers of P 2p_3/2_ and As 3d_5/2_ peaks provided. Raw data given as a solid-colored line, the cumulative fit as a dashed black line, and the background as a dashed gray line. Additional XPS is provided in SI, Figure S3.

An alternative approach to modifying layered materials is via alloying, as is well established for transition metal dichalcogendides.^6^ This approach may be accomplished for bP alloys via alloying phosphorus with its group 15 neighbor, arsenic. By partial substitution of phosphorus with arsenic precursors in typical bP syntheses, so-called black AsP (bAsP) is formed with the orthorhombic puckered honeycomb lattice structure of bP, but with a fraction (*x*) of the P atoms replaced by As atoms (Figure 1a) over a continuum of As:P ratios.^7^ The introduction of arsenic modifies the properties of bP, for example, by increasing anisotropic thermal conductivity^8^ and improving stability in air.^9^ Crystals of bAsP are most commonly synthesized by mineralizer-assisted (Sn/SnI_4_ or Au/Pb/PbI_2_) vapor transport from red phosphorus and gray arsenic^1,10^ although mineralizer-free^11^ and other syntheses, such as molecular beam deposition,^12^ are in development. The properties of bAsP depend on the elemental ratio and, in theory, the distribution of atoms within the structure, although current syntheses are all assumed to form stochastic As/P distributions. We note that an upper bound of *x* = 0.83 has been proposed by Osters et al.,^10^ with higher As contents stabilized by impurities or converting to the gray arsenic structure, although allegedly pure bAs is commercially available and has been used in multiple devices to date.^8^

The formation energies of differing bAsP atomic arrangements for any given stoichiometry have been calculated to predominantly exist within 0.17 eV (∼16 kJ mol^–1^) of the lowest energy configuration, invariant of the stoichiometry. Similarly, while the in-plane unit cell volume increases significantly with the As fraction from the outward displacement of As versus P, the volume remains near constant between most local configurations of each stoichiometry, although a small fraction of configurations have large lattice constant fluctuations.^13^ With increasing *x*, the band gap of the bulk materials decreases^14^ from 0.33 eV of bP to 0.15 eV for *x* = 0.83. This band gap range has made bAsP particularly promising for room temperature mid-to-long-wave infrared photodectectors^15−17^ and modulators.^16^

The exfoliation of bAsP into monolayer “AsPene” (or few-layered analogues) through typical layered-crystal exfoliation processes, such as the scotch tape method^1^ or sonication in solvents,^18^ allows for greater tuneability of the bAsP properties, with modification to the direct band gap. Further, AsPene has been theorized to have charge mobilities^19^ of up to 14,380 cm^2^ V^–1^ s^–1^, ballistic transport down the zigzag axis,^20^ and superconductivity^21^ (*T*_c_ = 21 K at 22 GPa). Beyond, AsPene has been proposed as a next generation material for microheaters,^22^ thermoelectrics,^8^ field effect transistors,^20,23^ solar cells,^19^ gas sensors,^24^ all-optical logic devices,^25^ and batteries,^7,26,27^ although akin to phosphorene, it degrades with oxygen and water vapor.^28,29^

The potential of phosphorene has been dramatically expanded recently with the bulk synthesis of quasi-1D phosphorene nanoribbons^30^ (PNRs) in 2019. The creation of nanoribbons is a well-established route to controlling and diversifying the properties of a parent 2D nanomaterial by introducing both additional quantum confinement and a large fraction of edge-states, providing control over the band structure and opening a range of exotic solid-state phenomena. Nanoribbon analogues of many nanomaterials are now well developed including graphene,^31^ transition metal dichalcogenides (TMDs),^32−34^ and MXenes.^35^ Several PNR syntheses have been developed,^36^ including lithography of phosphorene sheets^37^ and chemical vapor deposition (CVD)^38^ which gives wide, many-layer PNRs; future improvements to CVD syntheses may build on the developments in epitaxial CVD of TMD nanoribbons.^32−34^ The smallest width PNRs to date, i.e., with the most significant lateral confinement and deviation from 2D phosphorene-like properties, were synthesized through the lithium electride reduction (Li/NH_3(*l*)_) of bP,^30^ or rapid electrochemical sodium intercalation.^39^ The electrochemical sodium intercalation route forms ribbons by degrading bP preferentially down the bP corrugation axis, with the residual nondegraded material consisting of PNRs encased in bulk metal phosphides. Conversely, the electride route forms LiP_*x*_ intercalation compounds, which contain weaker P–P bonds connecting zigzag edges along the intralayer corrugations than in unintercalated bP.^40^ In amidic solvents, the intercalated material can dissolve spontaneously as PNR anions. The resultant PNRs cut exclusively parallel to the corrugations to give atomically straight edges along the zigzag axis, with widths and lengths of 4–50 nm and 0.1–1 μm, respectively. The early experimental work confirms the remarkable promise of PNRs, including improving the stability of lithium battery anodes,^41^ and improving photovoltaics through enhanced hole conductivity.^42^ Additionally, the as-synthesized anionic PNRs have recently been shown to exhibit room temperature magnetism attributed to edge-localized charges along the semiconducting species.^43^

In contrast to the significant body of PNR theory which proceeded their bulk synthesis, comparatively little work has been undertaken into the properties of bAsP nanoribbons (AsPNRs). It has been proposed that the zigzag edge states of AsPNRs have a magnetic moment (∼1 μ_B_) with the ferromagnetic state and the intraedge antiferromagnetic state being close in energy and similar in band structure, while armchair edges are expected to reconstruct into a nonmagnetic state.^44^ The optical properties have been predicted to vary significantly with As/P stoichiometry and distribution, with most configurations of very narrow AsPNRs expecting direct band gap semiconductors around 0.9 eV, while some configurations provide indirect gaps.^45^

Here, AsPNRs have been synthesized by extending the lithium electride PNR synthesis to bAsP, which are incorporated into two devices to demonstrate and measure their properties.

## Results and Discussion

A commercial bAsP alloy (*x* = 0.45) was used for AsPNR formation, which was shown to contain tin iodide in the XPS (Supporting Information, SI, Figure S3), indicative of its synthesis via the SnI_4_ mineralization procedure. The averaged Raman spectra (Figure 1di, *N* = 121) of the initial bAsP showed the three prominent bP A_g_^1^, B_2g_, and A_g_^2^ modes at 360, 435, and 462.5 cm^–1^, respectively, downshifted from pure bP from arsenic doping and bond lengthening. The broader overlapping bAs-derived A_g_^1^, B_2g_, and A_g_^2^ are also present (∼226, 238, and 254 cm^–1^), as well as a multitude of intermediate As–P peaks which have been observed previously in As_*x*_P_(1–*x*)_ Raman spectra,^26^ e.g., a small separate mode at ∼321 cm^–1^. Notably, the bAsP displays a distinct range of Raman spectra at different local points on the same sample, indicative of the wide distribution of local As/P stoichiometries arising from the stochastic nature of As and P within the sample (SI, Figure S4). The distribution of structures can also be seen in the powder X-ray diffraction pattern (pXRD, Figure 1b) where peaks are broadened versus those of typical bP crystals. The center of the broad (020) peak at 2θ = 16.52° indicates an interlayer spacing of 5.42 Å, but full indexing is complicated by the differing local arrangements of the framework atoms. Density functional theory (DFT) calculated models of layered bAsP of differing arrangements at the same As/P stoichiometry have formation energies within 0.026 eV/atom and unit cell volumes within 3% of each other (consistent with the 2D AsPene models of Sun et al.^13^) but show varied predicted pXRD patterns (SI, Section 2).

The bAsP was subjected to reduction with a lithium electride ammonia solution for 16 h, forming lithium intercalation compounds. The lithium ratio was defined versus a weight-averaged AsP atom (*M*_w_ = 50.75), and three stoichiometries were used to create a range of intercalation compounds: Li(AsP)_4.5_, Li(AsP)_9_, and Li(AsP)_18_. The pXRD of Li(AsP)_9_ shows the lithium intercalates successfully, seen as a significant downshift of all peaks, with the (020) peak maximum center 2θ shifting to 16.26°, implying an interlayer increase to 5.50 Å (Figure 1b). The general peak pattern is retained indicating that the general AsP framework structure is maintained. The lithium content changed the appearance of the residual material, with an excess red residue present on Li(AsP)_4.5_, orange-tinged Li(AsP)_9_, and limited discoloration on the most lithium-poor Li(AsP)_18_. Upon charging, the Raman modes broadened significantly and downshifted (Figure 1dii), as has been observed previously for intercalation of layered crystals with group 1 metals (e.g., bP, graphite) and is associated with a nonadiabatic renormalization of the phonon energies due to increased electron–phonon interactions,^46,47^ while the lower energy As modes became dominant. The spectra were reasonably consistent for all measured Li:AsP stoichiometries (SI, Figure S5), with minor variation in low energy peaks (∼200 and 180 cm^-1^) which are absent in Li(AsP)_18_ and most prominent in Li(AsP)_9_, which may tentatively be assigned to B_1g_ and B_3g_ phosphorene edge-modes from symmetry breaking.^48^ Reduction to Li(AsP)_9_ leads to the emergence of an additional low energy peak in the X-ray photoelectron spectroscopy (XPS) As 3d region at 39.1 eV, while the P 2p region remains unchanged from that of the bAsP (Figure 1e,f).

**Figure 2:**
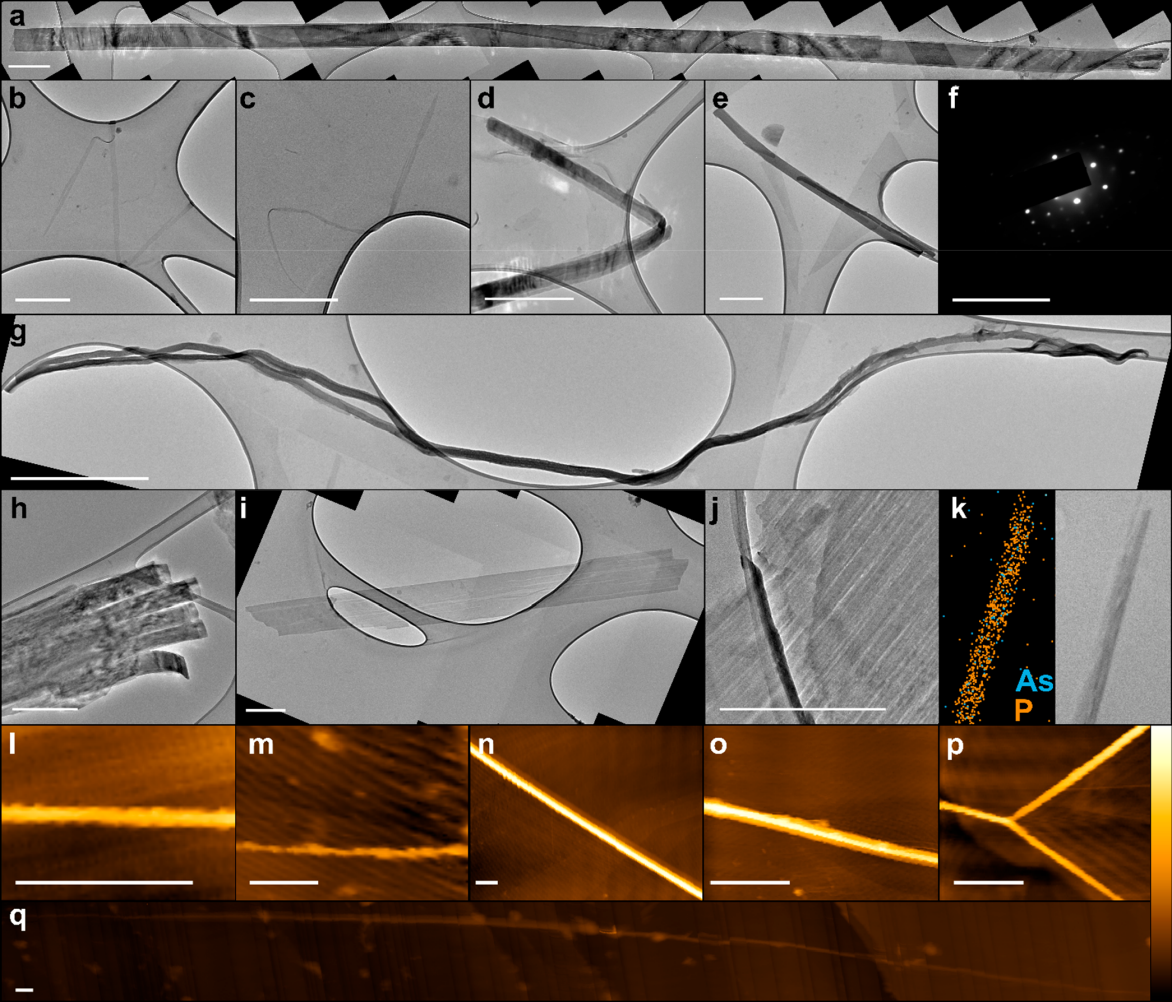
Microscopy of AsPNRs (a–e,g) TEM of AsPNRs, scale bars 500 nm; (f) selected area electron diffraction from region of ‘a’, scale bar 20 nm^–1^. (h) TEM micrograph of frayed large bundle end, (i,j) TEM micrographs of fractured 2D AsPene sheet, scale bar 500 nm. (k) EDS map of As/P with corresponding scanning TEM image; diffraction TEM micrograph of ribbon provided in SI, Figure S8t, (l–q) AFM micrographs of AsPNRs, *xy*-scale bar 1 μm, *z*-scale bar bottom right set between 0 nm and (l) 27.3 nm, (m) 40.8 nm, (n) 99.0 nm (o) 116 nm, (p) 76.2 nm, (q) 555 nm. Additional TEM micrographs and SAED provided in SI, Figures S8–10. Line cuts of AFM micrographs provided in SI, Figures S12–13.

The Li(AsP)_*y*_ salts could be dispersed in amidic solvents (Figure 1c), and the solutions were analyzed by drop-casting onto lacey carbon grids for transmission electron microscopy (TEM). The Li(AsP)_9_ showed AsPNRs (Figure 2) with some few-layered 2D species, while for Li(AsP)_18_ primarily ∼1 μm few-layered AsPene fragments were observed, alongside a very small number of AsPNRs (SI, Figure S6) and Li(AsP)_4.5_ formed predominantly small fractured species, with the excess of lithium thought to degrade the bAsP framework (SI, Figure S7).

The distribution of AsPNRs to 2D-AsP few-layered sheets formed from Li(AsP)_9_ was correlated with the solvent used for dispersion, with DMF leading to a significantly higher fraction of AsPNRs than NMP; the reason for this difference is not currently known. The ribbons showed a large range of lengths, with narrow (20–50 nm) few-layered ribbons several microns long (Figure 2b–c,l–m,q), dissolved alongside wider multilayer ribbons (Figure 2a,d–e,n–o), which often appear to have slid past each other to expose fewer-layer edges. In all cases, the ribbons were shown to be highly flexible, capable of bending around the lacey carbon framework, and to seamlessly traverse the step edges of the HOPG substrate in AFM (Figure 2q). Some of the longer multilayer ribbons formed bifurcations (Figure 2g,p) where the ribbon split into two separate thinner ribbons, with the total height of each branch matching the parent AsPNR (SI, Figure S12), forming seamless 3 semiconductor junctions. The ribbons are highly crystalline, as seen by selected area electron diffraction (SAED, Figure 2f) with reflections consistent with the orthorhombic structure of the parent bAsP, with in-plane unit cell values of *a* = 3.9 Å and *c* = 4.8 Å (SI, Figure S9). Concurrent rotation of the SAED with the rotation of a twisted ribbon in real space highlights the single-crystal character (SI, Figure S11).

While AsPNRs showed cuts primarily along the zigzag axis, they lacked the crystallographically straight edges of PNRs from lithium electride reduction of bP, with rough edges which varied between layers of few-layered ribbons, occasionally seen as splaying of one ribbon away from the stack. The ends of some larger ribbons also show fraying of smaller constituent ribbons (Figure 2g), although the single-crystal character further down the ribbons implies that these derive from incompletely propagated cracks. This in-plane fracturing divergent from the zigzag axis is also seen in the 2D AsPene sheets (Figure 2h,i) which show acute, nonparallel propagating cracks. The change from the straight fracturing of phosphorus-only PNRs may intuitively be understood to arise from the stochastic distribution of As/P atoms in bAsP. The diversion of propagating cracks leads to a lower quality of edges, which will likely influence the final AsPNR properties. Two rationalizations are proposed: the randomized structure will lead to local in-plane strain from the subtly different sizes of adjacent As_*x*_P_(1–*x*)_ units of differing *x*,^13^ with the asymmetric strain deflecting propagating cracks. Alternatively, it is possible that As-rich regions may be less chemically stable to reduction than P-rich regions (as supported by the preferential doping seen in XPS, Figure 1f,g), forming amorphous arsenides that deflect growing cracks. A more uniform distribution of As/P would be expected to mitigate both proposed effects to grant straight ribbon edges in the future.

**Figure 3:**
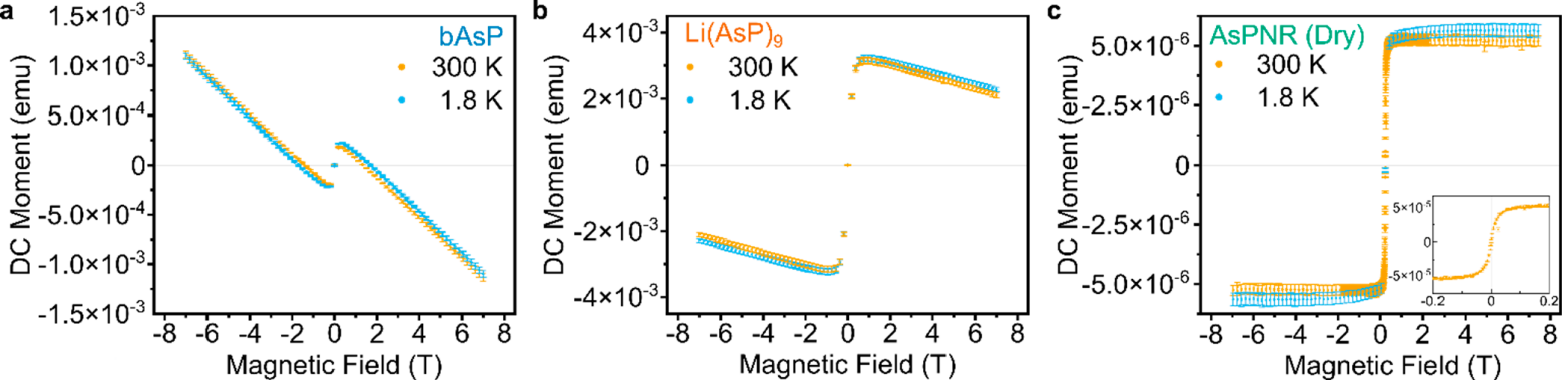
SQUID isothermal magnetic field sweep at 300 and 1.8 K of (a) AsP, (b) Li(AsP)_9_, and (c) solid AsPNRs drop cast from DMF solution. N.B, slower scans for AsPNR (dry) were performed between −2 and 2 T to provide high resolution around the zero-field point of 300 K, as shown inset between −0.2 and 0.2 T with y-scale between −0.7 × 10^–5^ and 6 × 10^–5^ emu with 0 T and 0 emu highlighted with gray lines. Additional data provided in SI, Figures S14–16.

The magnetic properties of the materials through the reaction process were interrogated using superconducting quantum interference device (SQUID) static magnetization measurements. The initial unintercalated bAsP is predominantly diamagnetic, with a small paramagnetic response that quickly becomes diamagnetic above small applied fields (Figure 3a). The residual weak magnetism is presently unassigned, although metal impurities in the bAsP framework may contribute a small paramagnetic signal.^49^ The behavior changes dramatically with intercalation to Li(AsP)_9_, where the material is much more strongly paramagnetic at low magnetic fields, at both 1.8 and 300 K (Figure 3b), with a significantly weaker diamagnetic component. The temperature-dependent magnetic susceptibility of the Li(AsP)_9_ in a 50 mT field shows a sharp drop below 3.9 K which is not present in the initial bAsP (SI, Figure S14). While other layered materials including bP have shown superconductivity with s-block metal intercalation,^50,51^ the presence of tin(IV) iodide in our commercial bAsP (SI Figure S3) being reduced to superconducting Sn^0^ is a more likely explanation, as rationalized previously for Li/bP.^52^

Solid-state AsPNRs drop-cast from DMF solutions (selected due to the higher propensity to form ribbons) show significantly different behavior from the paramagnetic Li(AsP)_9_, with no diamagnetic component and a vanishing coercive field, typical of small-domain nanomagnets, attributed to the formation of edge-states. We have recently measured similar behaviors in the related PNRs,^43^ although the atypical ferromagnetic-like hysteresis of PNRs does not appear to be present here, possibly due to the lack of the crystallographically clean, zigzag-only edges. The SQUID magnetometry also provides insight into the mechanism of formation of AsPNRs; the persistence of a diamagnetic component in Li(AsP)_9_ implies that it does not contain individual ribbons (stacked or encased in amorphous lithium phosphide/arsenide) but 2D sheets, which are then cut into paramagnetic ribbons during exfoliation.

**Figure 4:**
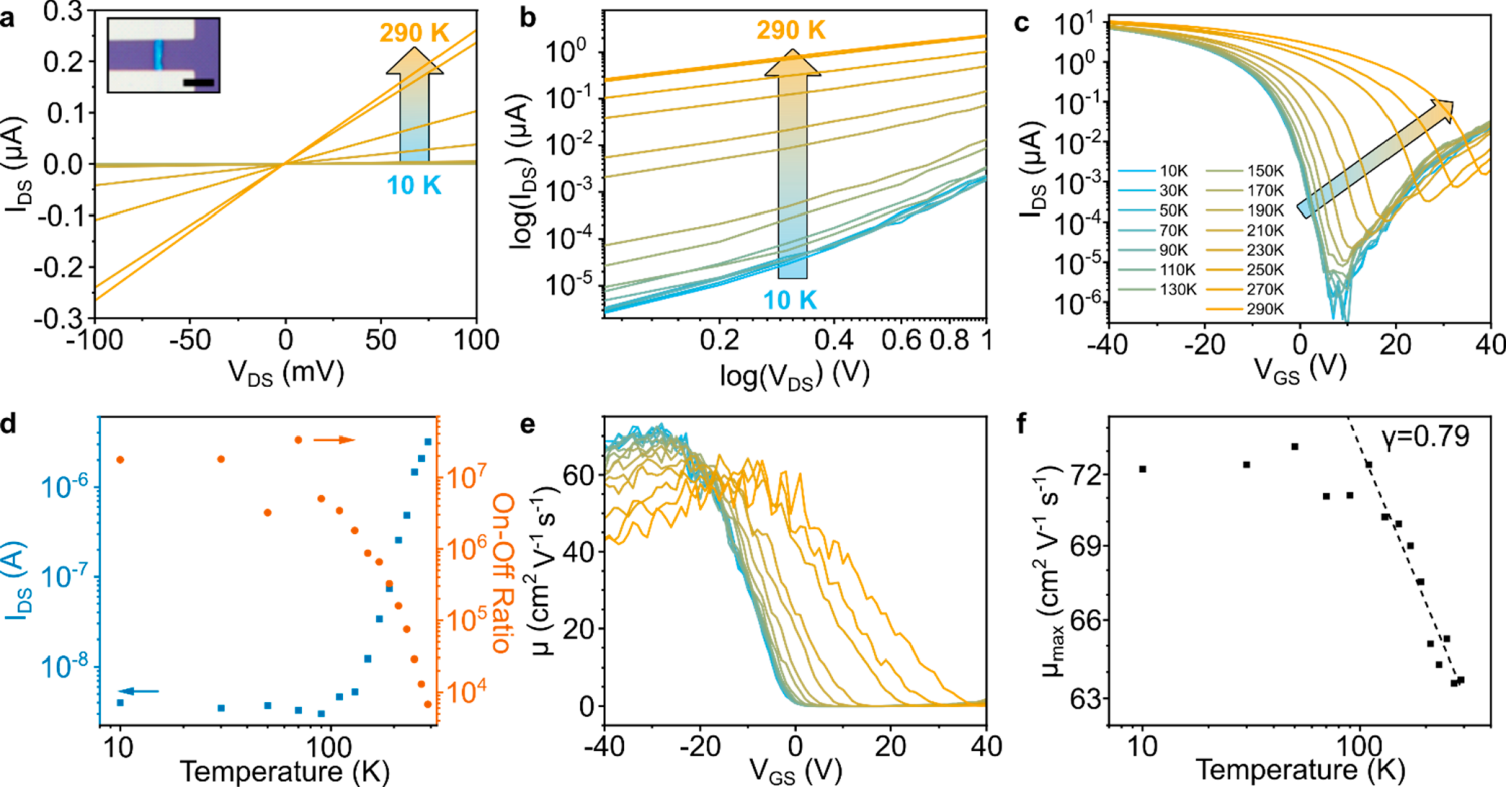
Field effect transistor (FET) characteristics of AsPNR device. (a) Temperature dependent FET output curves of an AsPNR FET at V_GS_ = 0 V between 10–290 K, and an optical image of the AsPNR device inset (scale bar of 5 μm). Wider voltage range provided in SI Figure S18. (b) The log-scaled curves at the source–drain current range of 0.1 V < *V*_DS_ < 1 V, indicating the current level is rising at *T* > 130 K. (c) Semilog transfer curves for temperatures ranging from 10 to 290 K. (d) Source–drain current (*I*_DS_) at zero bias (blue) and a temperature-dependent on–off ratio of the device (orange). (e) Temperature dependent field-effect mobility. (f) Temperature dependent maximum field-effect mobility showing power law dependency above 130 K (μ α T^–γ^).

To investigate the electronic properties of the AsPNRs, field effect transistors (FETs) were assembled by drop-casting diluted DMF solution on SiO_2_ and evaporating gold contacts along the ribbon length, with good electrical contact confirmed by the Ohmic-like near-linear behavior of the *I*_DS_–*V*_DS_ curve at *V*_GS_ = 0 V (Figure 4a). Given the expected small-band-gap behavior of the AsPNRs, measurements were taken between 10 and 290 K (Figure 4a–b) and at temperatures above 130 K, the conductivity rises (Figure 4d). Below this threshold, the device shows ambipolar behavior (Figure 4c) including on–off ratio of 10^7^. However, as the temperature rises above 130 K, the on–off ratio decreases, accompanied by an increased on-current (Figure 4d); this behavior is maintained at different *V*_GS_, and temperatures (SI, Figure S17), showing p-type behavior as the system approaches room temperature. The transition to a conducting state at 130 K equates to a narrow band gap of 0.035 eV from the Boltzmann equation, with conducting behavior at *T* > 130 K. The field-effect mobility (μ_FE_) was calculated^53^ from eq 1, where *L* and *w* are the length and width of the channel, V_DS_ is the drain voltage (1 V), *C*_*i*_ is normalized capacitance for 300 nm-thick SiO_2_ (11.5 nF cm^–2^), and *G*_*m*_ has been calculated from Figure 4c–d.
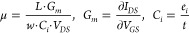
1

At low temperature, μ_FE_ was around 65 cm^2^ V^–1^ s^–1^ at *V*_GS_ < −20 V, and as the temperature increases above 130 K, the maximum μ_FE_ covered a wider range of *V*_GS_ (μ_FE_ is around 50.36 cm V^–1^ s^–1^ at *V*_GS_ = +2 V). By plotting the maximum μ_FE_ as a function of the temperature, we can gain insight into the charge scattering mechanism. The μ_FE_ appears to follow a power law dependence with temperature given by μ_FE_ α T^–γ^ with γ = 0.79 ± 0.046 at *T* > 130 K. The power law dependence with a positive exponent indicates a phonon scattering mechanism similar to other materials showing band-like transport such as graphene and transition metal dichalcogenides.^54^

This charge mobility of AsPNRs is expected to be one of its key properties, particularly under low bias, providing a room-temperature conducting analogue to the semiconducting PNRs.^2,36^ To confirm the utility of AsPNRs’ p-type hole mobility, a space-charge-limited-current (SCLC) hole-only device was assembled with our previously optimized configuration of indium tin oxide (ITO)/poly-3,4-ethylenedioxythiophene polystyrenesulfonate (PEDOT:PSS)/AsPNR/Au, alongside an AsPNR-free control device (ITO/PEDOT:PSS/PTAA/Au). The mobilities were extracted from the high voltage linear region using eq 2, where ε is the relative permittivity (3 for organic films) and *d* is film thickness. The hole conductivity was enhanced from 3.27 × 10^–4^ cm^2^ V^–1^ s^–1^ to 3.49 × 10^–4^ cm^2^ V^–1^ s^–1^ upon the introduction of the AsPNR layer.
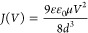
2

**Figure 5:**
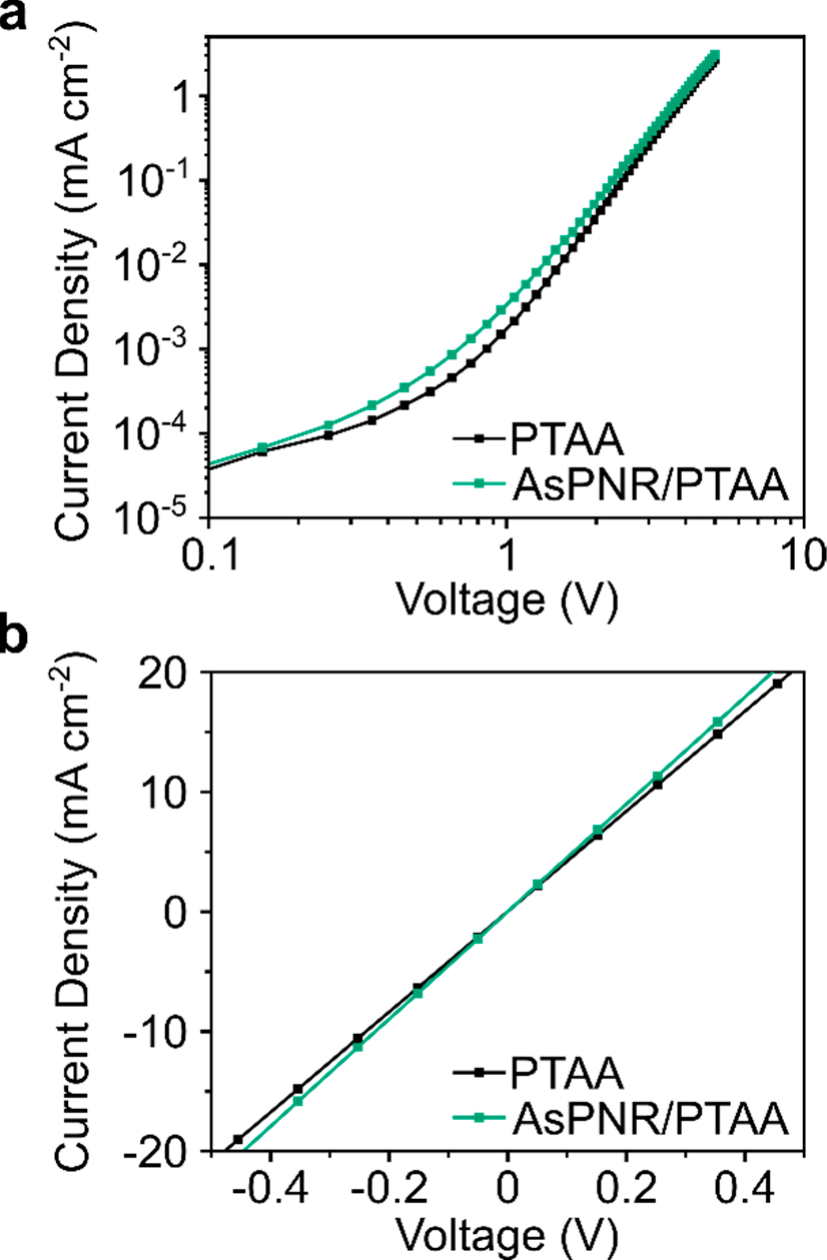
A log–log *J*–*V* plot of hole-only SCLC devices with the architectures ITO/PEDOT:PSS/PTAA/Au and ITO/PEDOT:PSS/PTAA/AsPNR/Au.

The direct current conductivity (σ_0_) was extracted from the slope of the current–voltage (*I–V*) curve, from eq 3, where *A* is the area of the sample and *d* is the thickness of the underlying hole transporting layer.^55^ The corresponding conductivities were extracted from Figure 5b as 4.033 × 10^–8^ S cm^–1^ (ITO/PTAA/Au) and 4.33 × 10^–8^ S cm^–1^ (ITO/PTAA/AsPNR/Au), which suggests that the presence of AsPNRs reduced the series resistance in the PTAA-only devices.^56^ It is expected that from the improved mobility and conductivity of devices incorporating the AsPNRs, charge carriers are more efficiently transported to the PTAA.
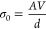
3

## Conclusions

The creation of AsPNRs has widened the breadth of group 15 quasi-1D nanoribbons by introducing alloyed species, which are expected to enhance the applicability and tunability of this new material family. In particular, the small band gap facilitates high conductivities above 130 K which will allow their use in applications where the low electrical conductivity of PNRs hinders their application, such as energy storage electrodes. Beyond, their small band gap, magnetism, and high hole mobilities make them ideal candidates for next generation near-infrared detectors, quantum computing, and charge carrier layers in solar cells, respectively. The bulk structural and transport properties of the alloy are well-defined, despite the local variation in As/P distribution. Improvements in synthesis of the parent bAsP are expected to improve the AsPNRs, in fine-tuning the As/P ratio, improving the uniformity of As/P distribution, and providing more crystallographically straight edges.

## Methods

### Materials

Bulk bAsP crystals were purchased from 2D Semiconductors Ltd. (Black AsP Alloy, As:P 0.6:0.4, 99.9995%). The material was sold as *x* = 0.6; however, XPS indicated *x* = 0.45 from the As 3d/P 2p regions (*N* = 3) and the most intense pXRD peak centers (2θ_(040)_ = 34.3°, 2θ_(113)+(020)_ = 40.8°) match *x* = 0.4 in the XRD series of Osters^10^ who grew crystals in *x* = 0.1 increments. Lithium metal (99.9995%, rod) DMF (99.8%, anhydrous), NMP (99.5%, anhydrous), and ammonia (≥99.98%, anhydrous) were purchased from Sigma-Aldrich, UK. Solvents were dried prior to use over activated 4 Å molecular sieves for >7 days. Ammonia was purified by condensation over excess lithium metal and transferred to a lecture bottle evacuated to ∼10^–7^ mbar prior to use.

### bAsP Intercalation

Bulk bAsP was outgassed at 100 °C under dynamic vacuum (∼10^–7^ mbar) in a glass tube for 3 days. In a high-purity argon glovebox (O_2_ and H_2_O < 0.1 ppm), the bAsP was lightly ground in an agate pestle and mortar and was added to a glass tube. Lithium metal was added to the outgassed crystal in a stoichiometric ratio of Li/AsP = 1/9 (unless specified). The glass tube was then attached to a leak proof, gas handling system and evacuated to ∼10^–7^ mbar. The tube was immersed in a propan-2-ol bath, and the temperature was cooled to −50 °C (Julabo FT902 chiller) and maintained at this temperature. High purity ammonia was slowly condensed onto the bAsP and lithium while the alkali metal was dissolved. For intercalation, the bAsP was left submerged in the dilute metal ammonia solution for 24 h, with the initial blue solution eventually turning light orange with the crystals clearly visible at the bottom. The system underwent cryo-pumping to remove and recover the ammonia, and the material was further dried under dynamic vacuum (<10^–5^ mbar for 1 h) leaving fractured crystals of Li(AsP)_*y*_ surrounded by pale orange residue. The intercalated sample was then removed from the bath and stored in an Ar glovebox. Photos of the procedure are provided in the SI, Figure S1.

### AsPene Nanoribbons

In the glovebox, Li(AsP)_*y*_ (typically ∼5 mg) was submerged in an anhydrous and aprotic solvent of choice (1 mL mg_LiAsP_^–1^, DMF or NMP) in a glass vial. The sample vials were sealed and placed in an ultrasonic bath (QS3, 50W) for 1 h to produce sonicated AsPNR dispersions and centrifuged (2000 rpm, 30 min, Hettich EBA 20 centrifuge) and decanted by hand to give a pale yellow solution of AsPNRs (Figure 2c, inset).

### pXRD

In an Ar glovebox, bAsP/Li(AsP)_9_ samples were lightly ground with an agate pestle and mortar before loading into a quartz capillary sealed with wax. Diffractograms (both capillary and acetate film) were recorded on a STOE SEIFERT (Cu kα = 1.5406 Å) at 2θ = 0.05° steps at 5 s per step. Attempts to measure pXRD of AsPNR cast/filtered from solution were unsuccessful, attributed to the submilligram quantities of AsPNRs.

### UV–vis Spectroscopy

In an Ar-filled glovebox, AsPNR solution in DMF was centrifuged (2000 rpm) and decanted into a quartz cuvette (*l* = 4 mm) fitted with a screw cap vial and rubber septum. The cuvette was closed and sealed with parafilm before removal from the glovebox. The sample was immediately measured with a Shimadzu UV-2600i at “medium” speed in 0.1 nm increments against an air background.

### XPS

The surface elemental composition of the samples was determined using a K-ALPHA Surface Analysis spectrometer (Thermo Scientific) equipped with an Al source (Kα = 1486.6 eV). bAsP and Li(AsP)_9_ were affixed using indium foil (99.999% purity, 0.5 mm thickness, Merck) to a sample plate, and AsPNRs were drop-cast from DMF solution onto a glass coverslip, and adhered to the sample plate with carbon tape. Li(AsP)_9_ and AsPNRs were loaded in a vacuum transfer sample holder (Thermo Scientific, 831-57-100-2) inside an Ar glovebox to ensure air-free sampling. The spot size used was 400 μm. Binding energies were calibrated to the C 1s advantageous carbon peak at 284.8 eV, with calibration for bAsP and Li(AsP)_9_ validated by comparison of In 3d fitting. Data was fitted with CasaXPS (v.2.3.25PR1.0) using a Shirley background for all regions except I 3d where a linear background was necessary. Spin–orbit splitting was fixed during fitting (P 2p = 0.84 eV, As 3d = 0.64 eV, Sn 3d = 8.41 eV, I 3d = 11.50 eV).

### Raman Spectroscopy

bAsP was measured after grinding, Li(AsP)_9_ was sealed in a quartz capillary in an Ar glovebox with wax, and AsPNR solution was deposited on a 5 × 5 mm^2^ silicon wafer through ten iterations of depositing 10 μL of DMF solution in an Ar glovebox, before drying in the vacuum antechamber at room temperature for 10 min. Raman spectra were recorded with a Renishaw InVia Raman spectrometer using a 785 nm laser, recorded at 5 mW power for 90 s using 1200 line/nm grating, with a measurement center at 400 cm^–1^. Measurements were taken over a 50 × 50 μm^2^ region of each sample, in a 11 × 11 square array of points with 5 μm separation (*N* = 121). Cosmic ray peaks were removed, and AsPNR data between 500–540 cm^–1^ were also removed to eliminate the dominant Si mode at 520.7 cm^–1^ (SI, Figure S4f). Data sets were normalized (SI, Figure S4a–c) and averaged.

### TEM

In an Ar glovebox, ∼50 μL of AsPNR solutions were drop-cast onto lacey carbon grids (200 mesh Cu support) placed on cellulose filter paper and left to dry overnight at room temperature. For DMF samples, the grid was loaded on the holder headpiece in a glovebox to prevent degradation during sample loading. The sample/loaded holder headpiece was sealed in glass vials for transport to the TEM and was loaded as quickly as possible in air before being evacuated in the TEM. Measurements were performed on a JEOL 2100 with an accelerating voltage of 200 keV. All samples recorded from DMF solution, except Figures 2f/S6/S7/S11 which were recorded from NMP solution.

### High Speed-Atomic Force Microscopy

AsPNR dispersions were drop-cast onto freshly cleaved highly ordered pyrolytic graphite (HOPG, GE Advanced Ceramics ZYH grade) in an Ar glovebox using a micropipette. The substrates were left overnight on a hot plate maintained at 70 °C. To remove NMP residue, samples were transferred to a custom-built apparatus for further drying in a turbomolecular pump. In this air-free system, the substrates were evacuated to 10^–6^ mbar and left under dynamic vacuum for 1 week before the temperature was increased to 100 °C for a further week. After heating, the samples were removed without exposure to air and stored in an Ar glovebox. All deposited AsPNRs were kept in an inert environment for a least 2 weeks before experiment. Contact mode high-speed AFM (Bristol Nano Dynamics Ltd., UK) was performed using silicon nitride cantilevers (Bruker MSNL, tip radius ranging from 2 to 12 nm). This technique allowed real-time video-rate imaging to locate and track the AsPNRs over their full multimicrometer lengths with nanometer pixel resolution in XY and subatomic resolution in Z. Images were typically collected at two frames per second with frame sizes up to 20 μm by 10 μm. These topography maps were then processed on Gwyddion^57^ to obtain their height and width distributions.

### DC Magnetization

Magnetization measurements were performed in a Quantum Design Ltd. Magnetic Properties Measurement System (QD MPMS). MPMS measurements of the magnetization of a sample utilize a Superconducting Quantum Interference Device (SQUID) magnetometer. DC magnetization measurements were obtained by mechanically moving a sample vertically through a homogeneous region of magnetic field, of constant applied magnetic field strength, using a fixed center. Measurements were performed over the temperature interval 1.8–300 K, at various applied magnetic field strengths up to ±7 T. Data for isothermal magnetic field scans were obtained by warming from 1.8 K, having previously cooled in zero field. After stabilizing at 1.8 K, the temperature was then increased, while applying a constant magnetic field strength of 50 mT. Prior to beginning any new magnetic field or temperature scan the system was equilibrated at 300 K, and the magnet was reset in order to remove any stray flux that could be trapped in the SQUID. Care was taken to ensure that samples were not touched by any magnetic material throughout the mounting and loading procedure, which involved securing sample capsules within a straw sample holder, sealed by Kapton tape. Samples were prepared in an argon glovebox, as crystals (∼1–2 mg, bAsP/Li(AsP)_9_), as DMF solution, or repeatedly drop-cast in one-half of a capsule from DMF solution (≪ 1 mg). Due to the submilligram quantities of AsPNRs used, values are presented without normalization to weight. Samples were transferred to the SQUID from the glovebox in sealed vials and were mounted as quickly as possible (∼1–5 min). For each measurement, multiple DC magnetization measurements were averaged, in order to yield a reliable measure of the bulk magnetization of the samples. Error bars are the standard deviation from averaging these multiple measurements.

### FET Measurements

AsPNR FETs were fabricated on prepatterned SiO_2_ (300 nm)/Si substrate. AsPNR DMF solution was drop-casted on the substrate, which was followed by electron-beam lithography for defining the electrodes. A 50-nm-thick Au layer was deposited as electrodes. Electrical transport of the device was measured in a cryogenic vacuum probe station (<10^6^ Torr, Lakeshore) in the temperature range 10 to 290 K using a semiconductor parameter analyzer (Keithley 4200). The device in Figure 4 used *L* = 5 μm and *w* = 1.5 μm for eq 1.

### SCLC Measurements

ITO was ultrasonically cleaned in acetone, Milli-Q water, and isopropyl alcohol for 10 min in each solvent. The ITO was then dried with nitrogen and treated with oxygen plasma for 7 min. PEDOT:PSS (Hercules) was filtered and spin-coated onto ITO at 5000 rpm (acceleration of 4000 rpm) for 45 s and annealed for 30 min at 150 °C. A 300 nm thick layer of PTAA (Ossila 14000 mW, 50 mg mL^–1^ in toluene) was then spin-coated on top of the PEDOT:PSS at 2000 rpm (acceleration of 1000 rpm). For samples with AsPNRs, an AsPNR NMP solution was spin-coated on top of the PTAA at 4000 rpm (acceleration of 5000 rpm) for 30 s and immediately annealed at 100 °C for 30 min, followed by gentle vacuum (<1 mbar) to remove excess solvent. Finally, 100 nm of Au were thermally evaporated as a top contact at a base pressure of 5 × 10^–6^ mbar.

### Density Functional Calculations

Density functional theory (DFT) calculations were performed with CRYSTAL17 using the hybrid exchange B3LYP functional and all-electron split valence plus polarization basis set for Li^58^ and triple-ζ valence plus polarization basis sets for As and P.^59^ See Supporting Information Section 2 for fuller details. Theoretical pXRD patterns (SI, Figures S21–23) were calculated using VESTA (v.3.5.8).

## Supporting Information Available

Digital photographs of AsPNR synthesis steps, higher resolution pXRD data, additional XPS spectra, Raman spectra, TEM micrographs of Li(AsP)_*y*_ dispersed in amidic solvents, AFM line cuts, isothermal magnetic field sweeps of control samples, zero field cooled temperature dependent magnetization measurements, temperature dependent field-effect mobility of FETs and additional FETs, DFT calculations (PDF)
