# Supplementary material for: Production of Magnetic Arsenic–Phosphorus Alloy Nanoribbons with Small Band Gaps and High Hole Conductivities
Source: J Am Chem Soc. 2023 Aug 8;145(33):18286–95. doi: 10.1021/jacs.3c03230 (PMC10450688; doi:10.1021/jacs.3c03230)
Supplement: Supplementary file 1 — ja3c03230_si_001.pdf [file ja3c03230_si_001.pdf]

# Supplementary Information: Production of Magnetic Arsenic-Phosphorus Alloy Nanoribbons with Small Band Gaps and High Hole Conductivities

Feng Fei Zhang,[a,b] Eva Aw,[b] Alexander G. Eaton,[c] Rebecca R. C. Shutt,[b] Juhwan Lim,[c] Jung Ho Kim,[d] Thomas J. Macdonald,[e] Cesar III D. L. Reyes,[a] Arjun Ashoka,[c] Raj Pandya,[c] Oliver D. Payton,[f] Loren Picco,[f] Caroline E. Knapp,[a] Furio Corà,[a] Akshay Rao,[c] Christopher A. Howard,[b]* Adam J. Clancy[a,c]*

a. Department of Chemistry, University College London, London WC1E 6BT, UK

b. Department of Physics and Astronomy, University College London, London WC1E 6BT, UK

c. Cavendish Laboratory, Department of Physics University of Cambridge, Cambridge, CB3 0HE, UK

d. Department of Materials Science and Metallurgy, University of Cambridge, CB3 0FS, UK

e. School of Engineering and Materials Science, Queen Mary University of London, London E1 4NS, UK

f. Interface Analysis Centre, H. H. Wills Physics Laboratory, University of Bristol, Bristol, BS8 1TL, UK

## Section 2 – Supplementary Data

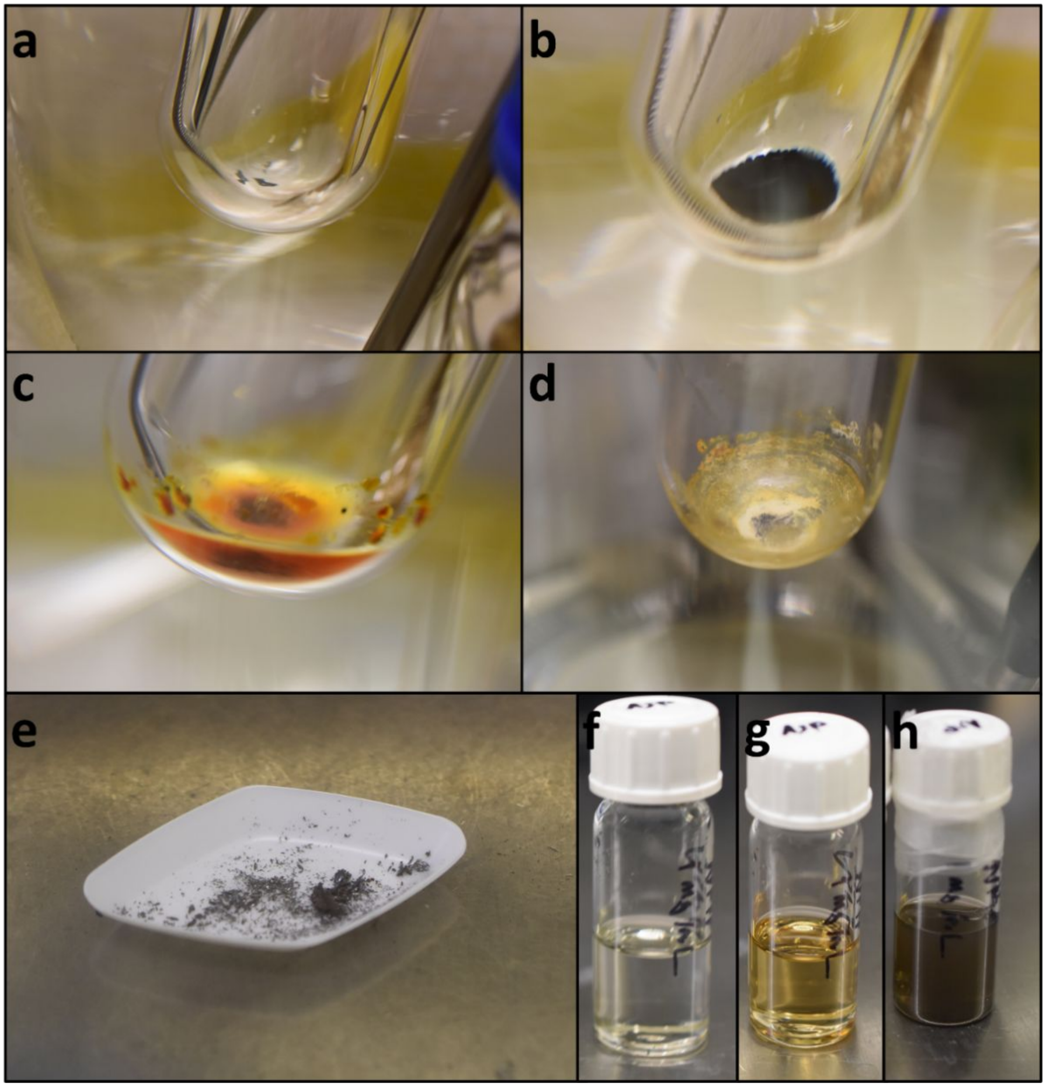

***Figure S1***. *Digital photographs of bAsP intercalation process in a glass tube submerged in a -50 °C IPA bath. (a) Li metal and bAsP crystals. (b) Condensation of liquid $NH_3$, forming deep blue solution from formation of lithium electride. (c) Mixture after 24h held at -50 °C showing orange solution attributed tentatively to formation of amorphous lithium phosphide/arsenide. (d) After evaporation of ammonia to leave black fractured crystals of $Li(AsP)_9$ surrounded by coffee rings of the impurity. (e) $Li(AsP)_8$ crystals in a weighing boat. (f) $Li(AsP)_9$ directly after adding NMP at 1 mg $mL^{-1}$ (g) $Li(AsP)_9$ in NMP at 1 mg $mL^{-1}$ after 1 week. (h) $Li(AsP)_9$ in NMP at 1 mg $mL^{-1}$ after 1 week soaking and 30 min bath sonication.*

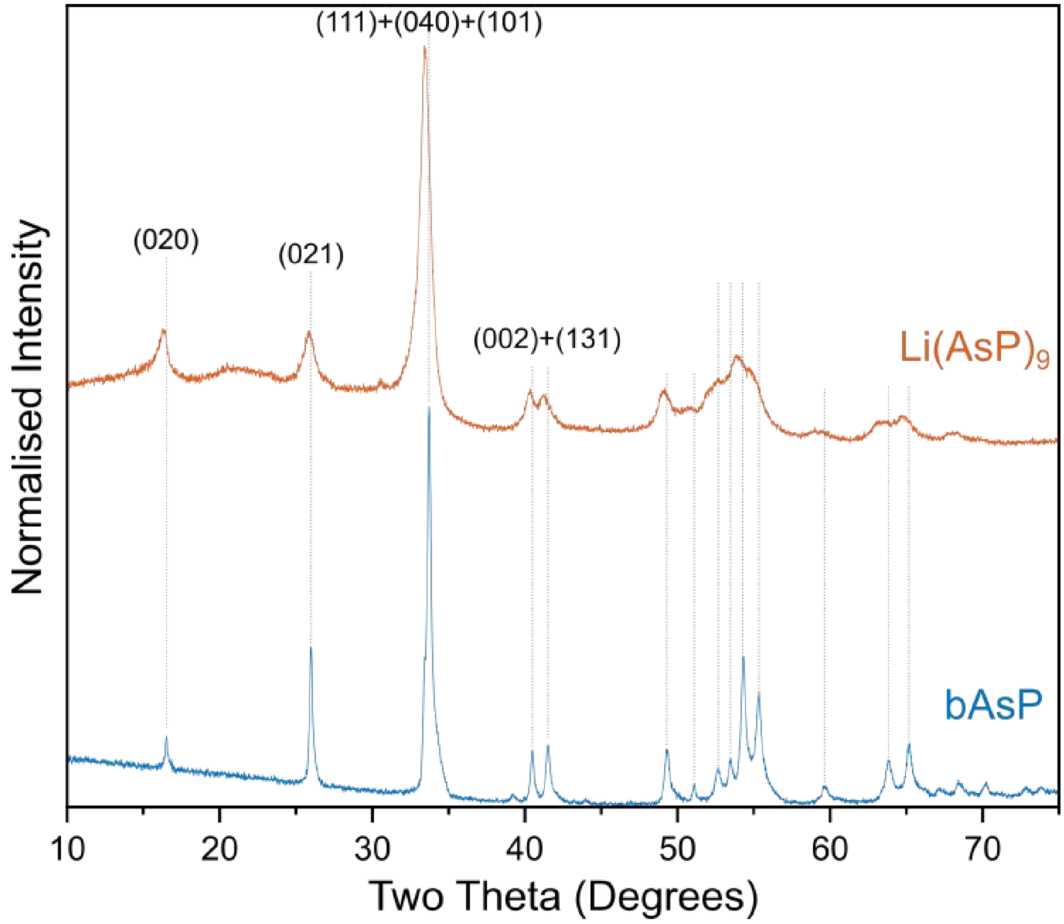


***Figure S2**. pXRD of bAsP (blue, bottom) and $LiAsP_9$ (vermillion, top). Larger replotting of main text Fig 1b, with dotted grey lines highlighting bAsP peak centres.*

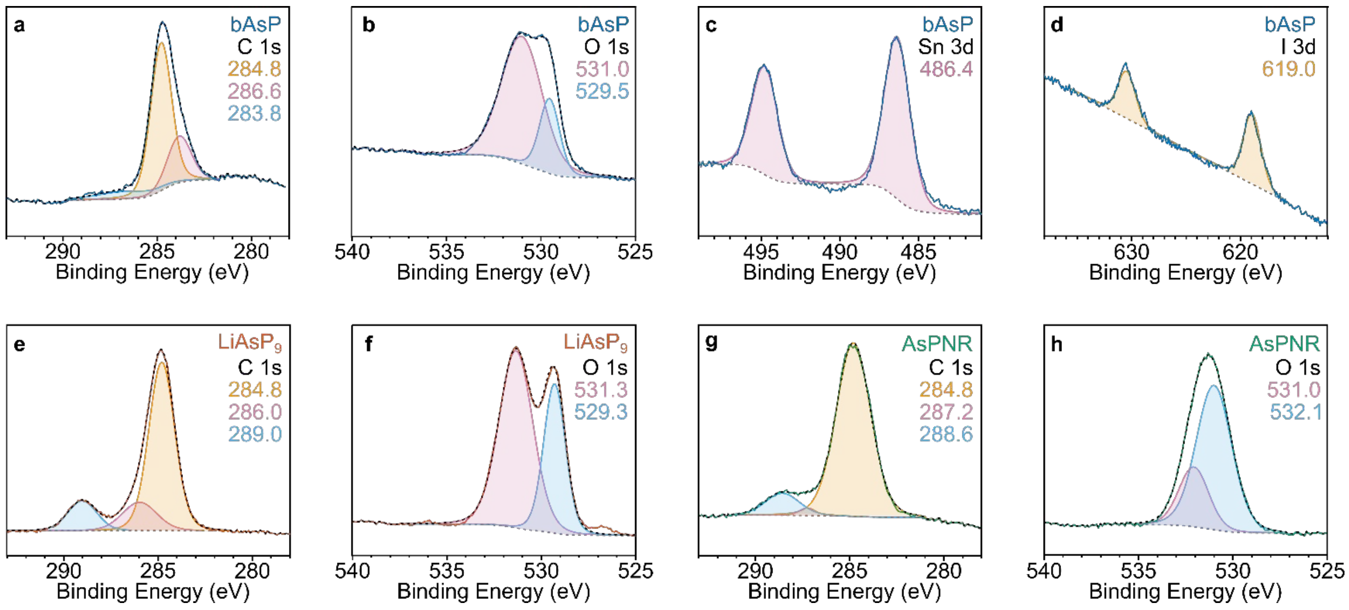


***Figure S3**. Additional XPS spectra. (a) bAsP C1s, (b) bAsP O 1s, (c) bAsP Sn 3d (d) bAsP I 3d (e) $Li(AsP)_9$ C 1s, (f) $Li(AsP)_9$ O 1s, (g) AsPNR C 1s, (h) AsPNR O 1s.*

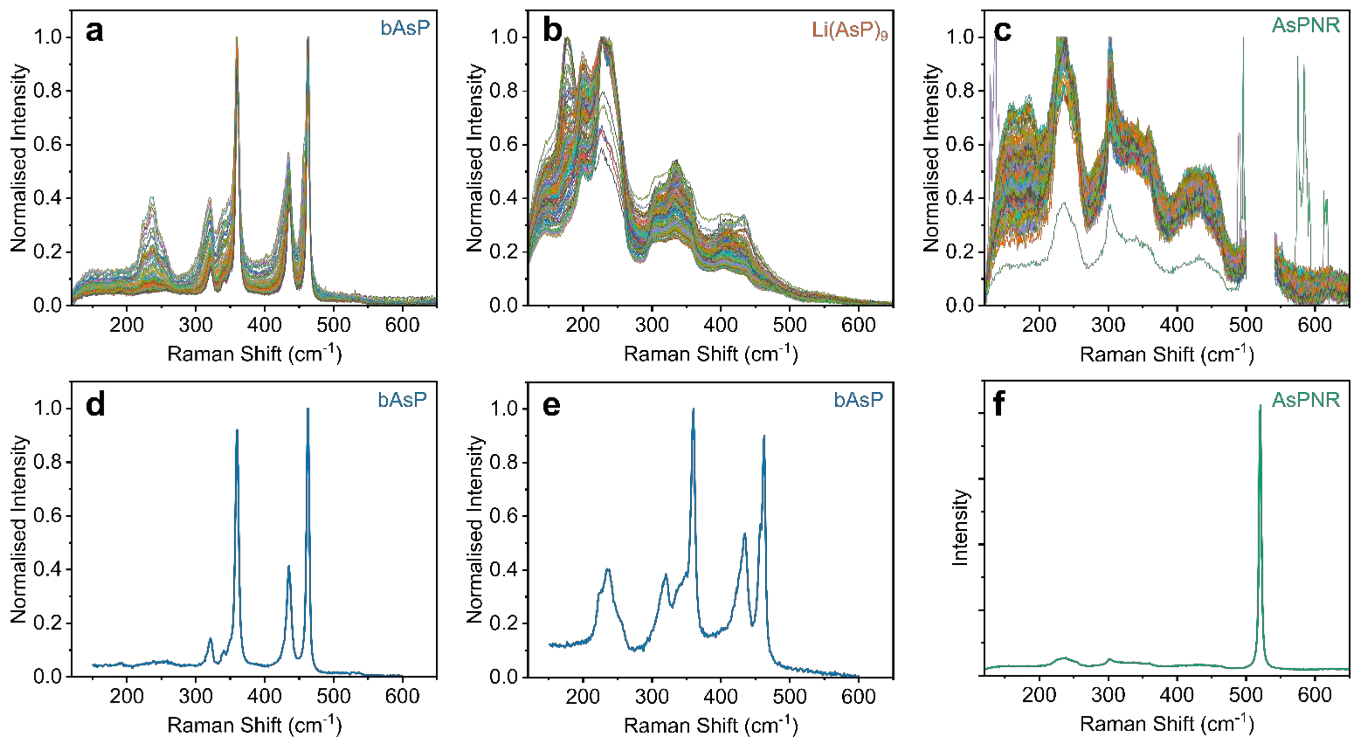


***Figure S4**. Additional Raman data. (a-c) Individual normalised Raman spectra from 50 × 50 μm square array recorded at 5 μm intervals for (a) bAsP, (b) $Li(AsP)_9$ and (c) AsPNR dropcast on Si wafer with 500 – 540 $cm^{-1}$ removed prior to normalisation due to Si peak at 520.7 $cm^{-1}$. (d,e) Individual spectra selected from 'a' to highlight (d) phosphorene-rich and (e) arsenene-rich regions within the bAsP initial material. (f) Raw Raman spectrum of AsPNR dropcast on Si to highlight the prominence of the Si mode.*

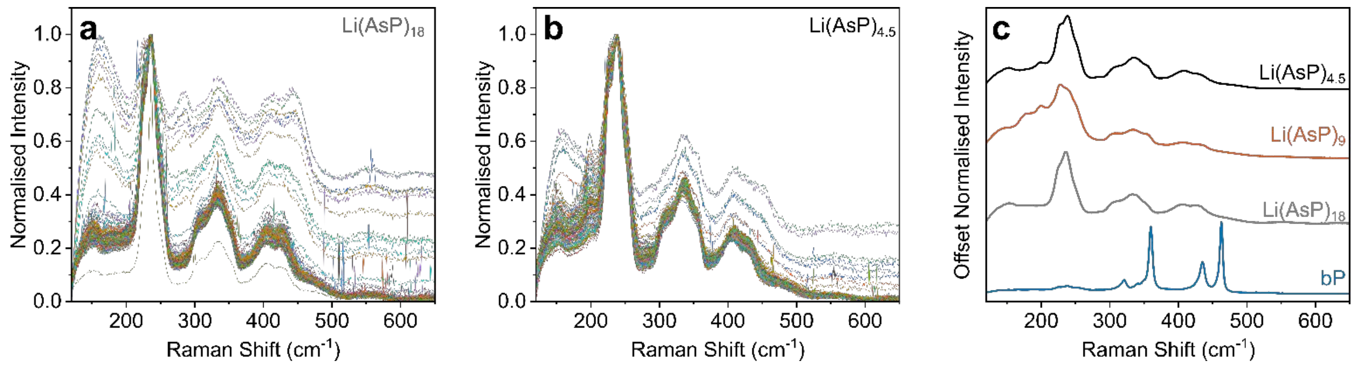


***Figure S5**. Supplementary Raman spectra (a) all mapped spectra of $Li(AsP)_{18}$, (b) all mapped spectra of $Li(AsP)_{4.5}$, (c) stacked averaged data of all $Li(AsP)_y$ stoichiometry.*

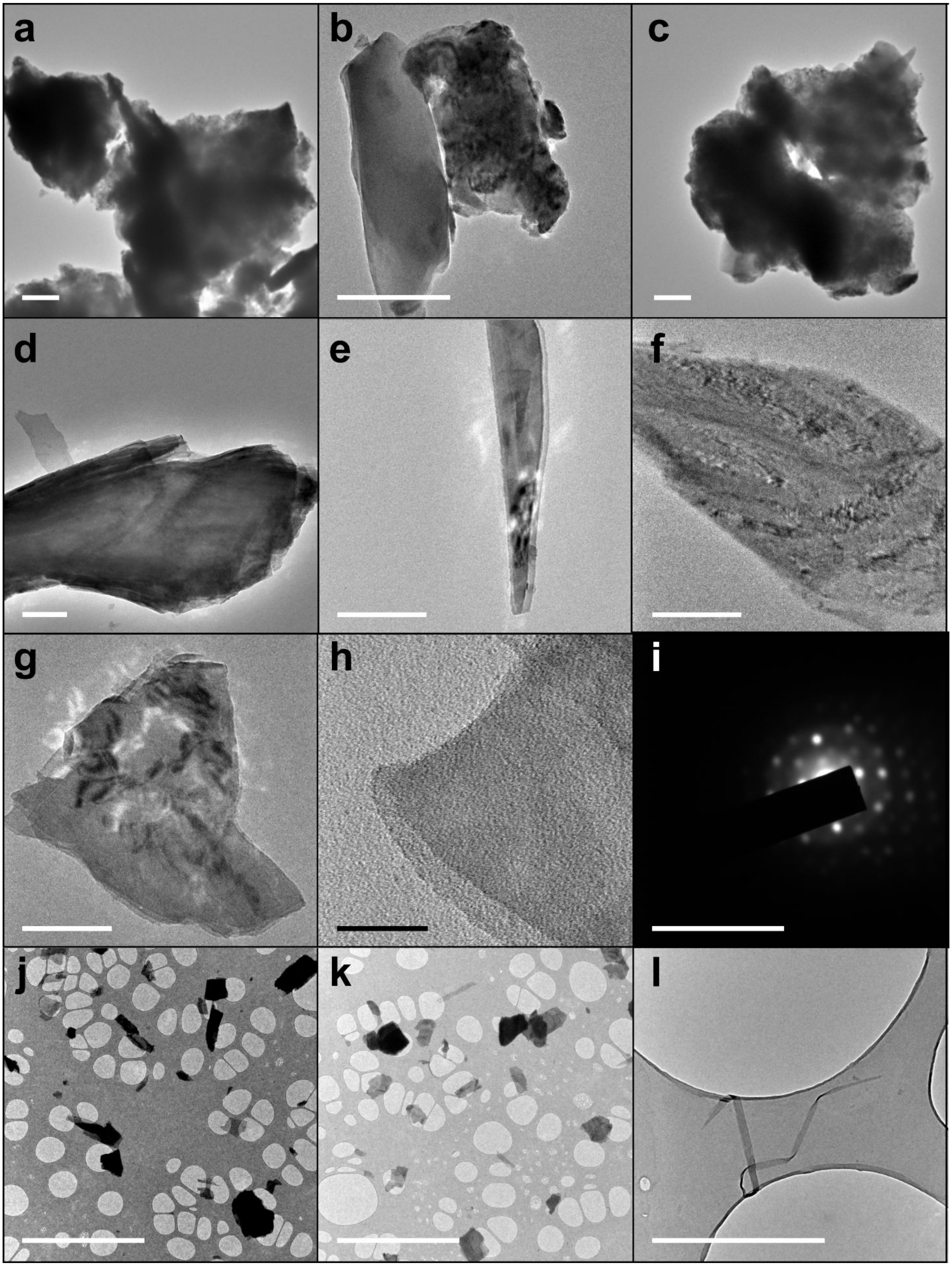


***Figure S6.*** *TEM micrographs Li(AsP)$_{18}$ (a-h) dispersed in NMP. (h) Magnified region of left corner of 'g', (i) SAED of h (j-l) Dispersed in DMF. Scale bars (a-g) 250 nm, (h) 20 nm, (i) 20 nm$^{-1}$, (j-k) 10 µm$^{1}$, (l) 1µm.*

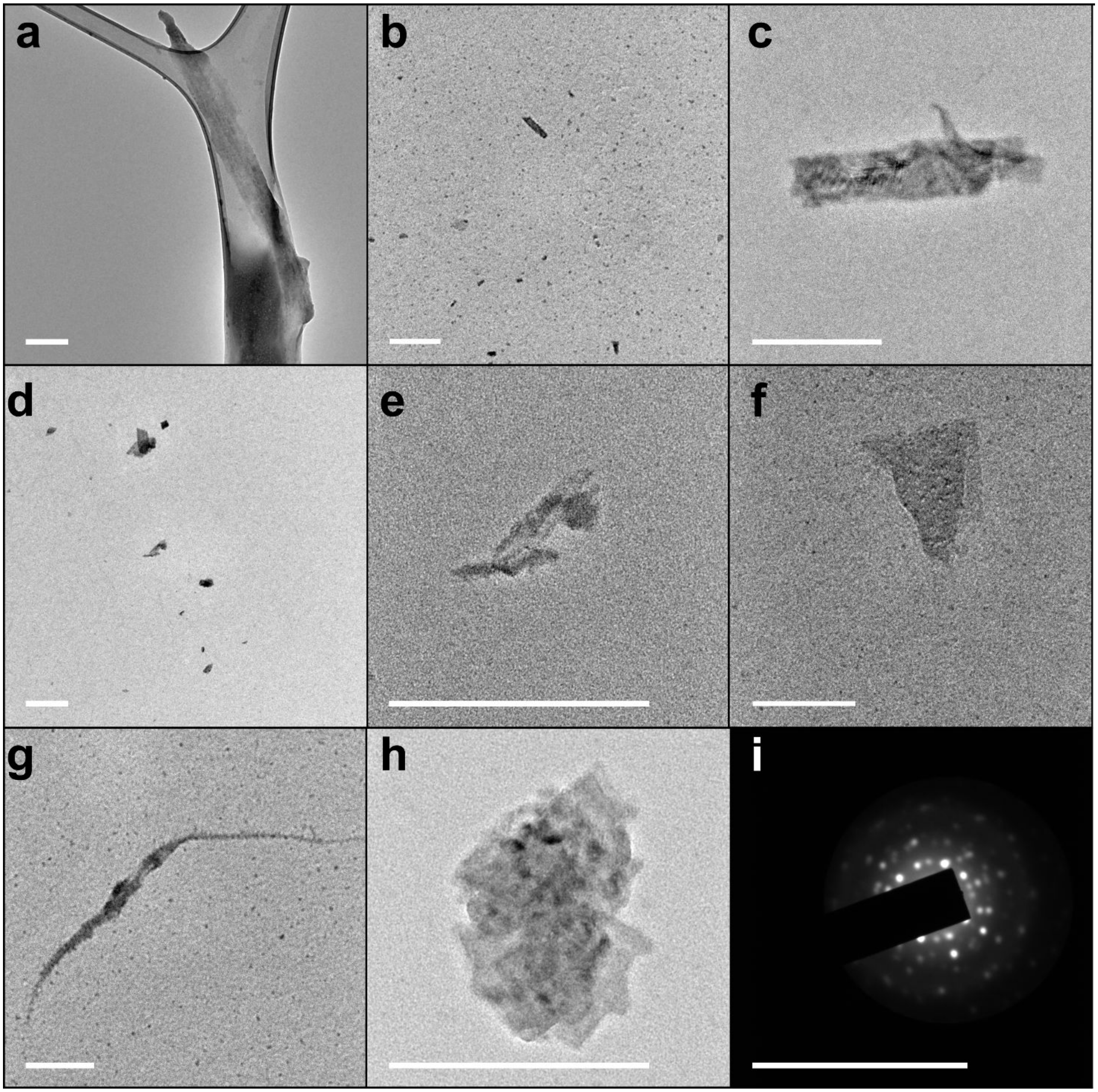


***Figure S7.*** *(a-h) TEM micrographs of Li(AsP)$_{4.5}$ dispersed in NMP. (i) SAED of 'h'. Scale bars (a-h) 250 nm, (i) 20 nm$^{-1}$.*

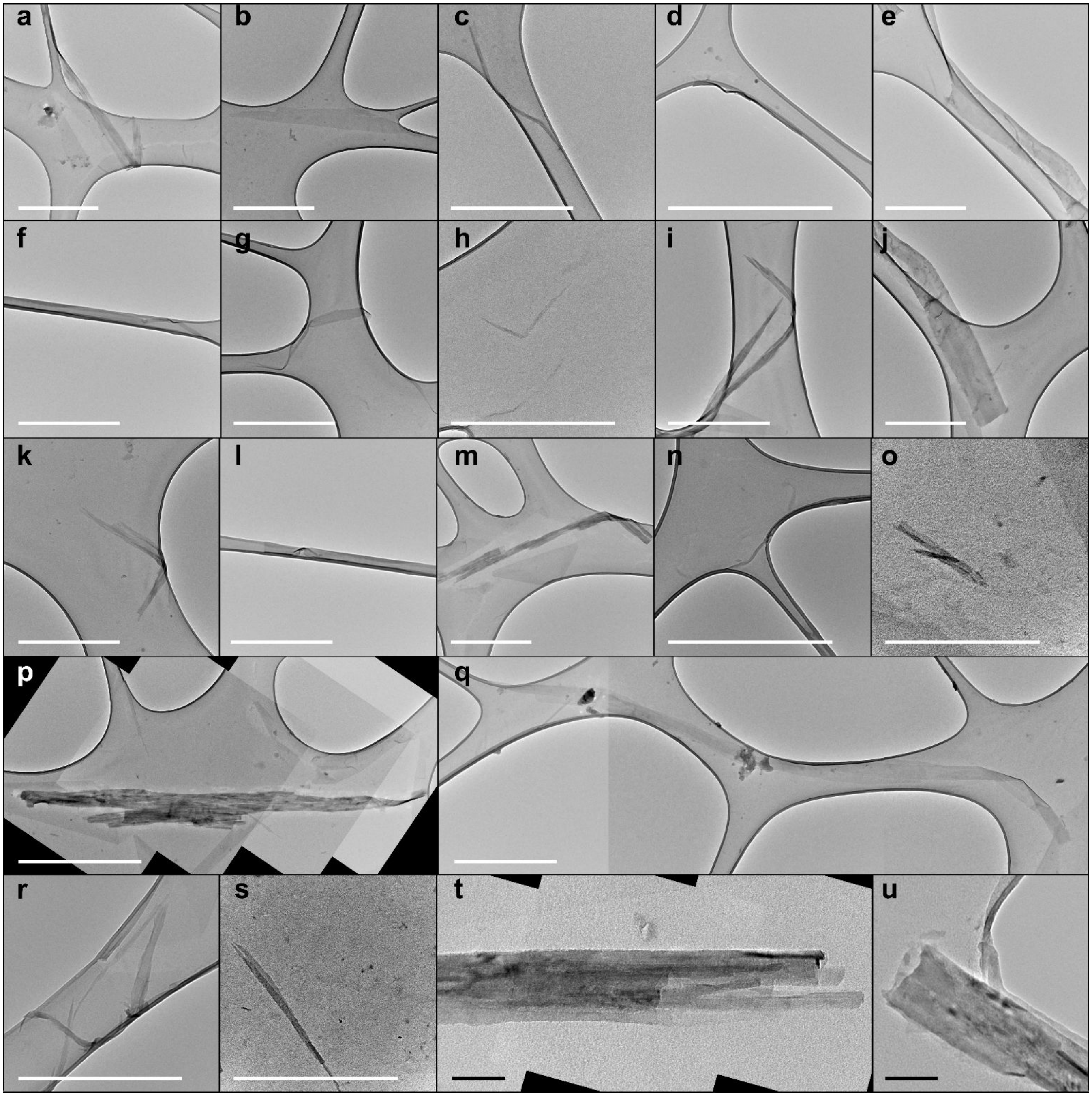


***Figure S8***. *Additional TEM micrographs of AsPNRs. Scale bars (a-s) 1 µm, (t-u) 100 nm.*

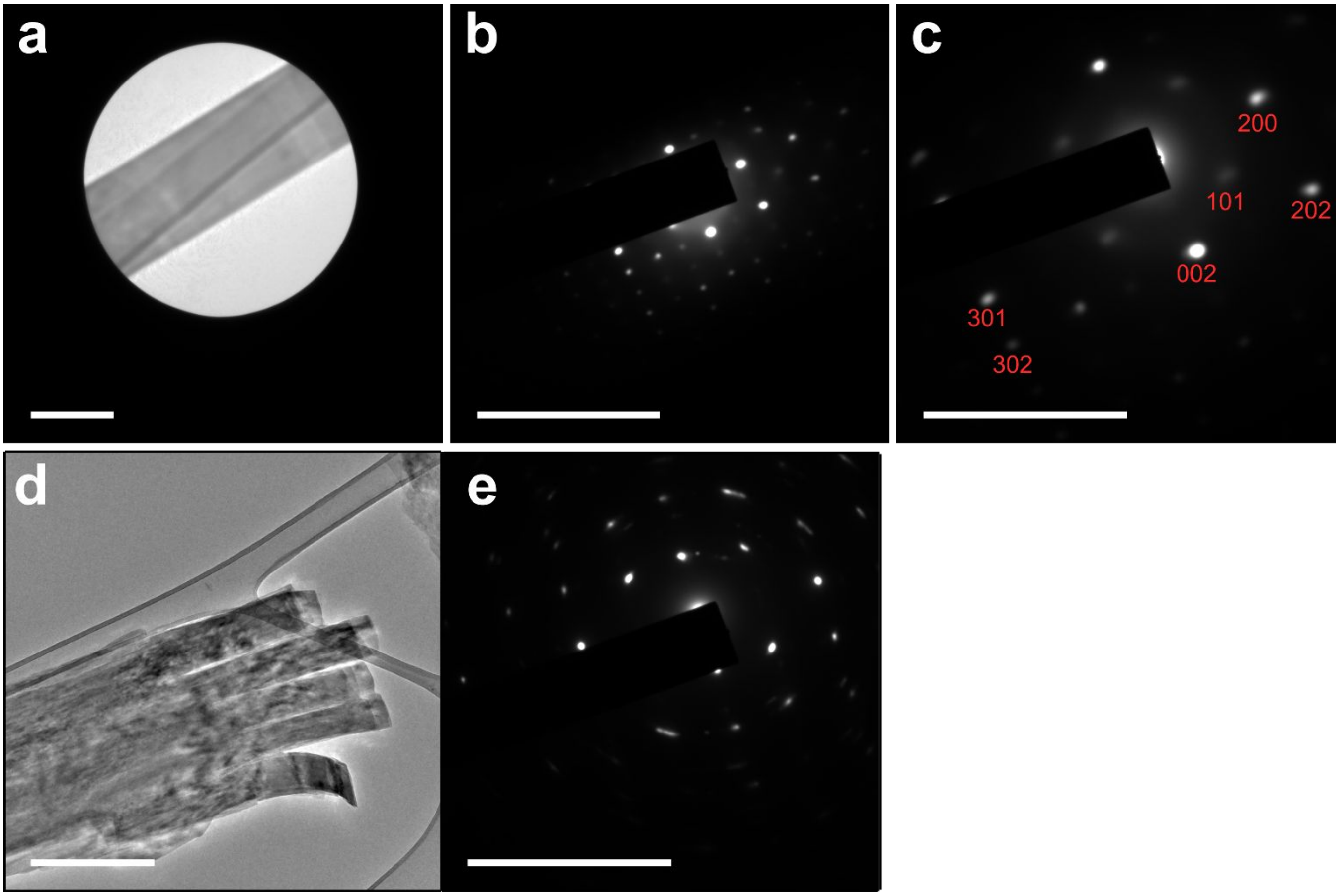


***Figure S9.*** *(a) Section of AsPNR from main text Fig. 2a under diffraction aperture used for main text Fig. 2e., scale bar 200 nm (b) reproduction of main text Fig. 2b SAED. scale bar 20 nm$^{-1}$ (c) Indexed higher magnification SAED 'b', scale bar 10 nm$^{-1}$ (d) reproduction of main text Fig. 2g, scale bar 500 nm (e) SAED of 'd', scale bar 10 nm$^{-1}$.*

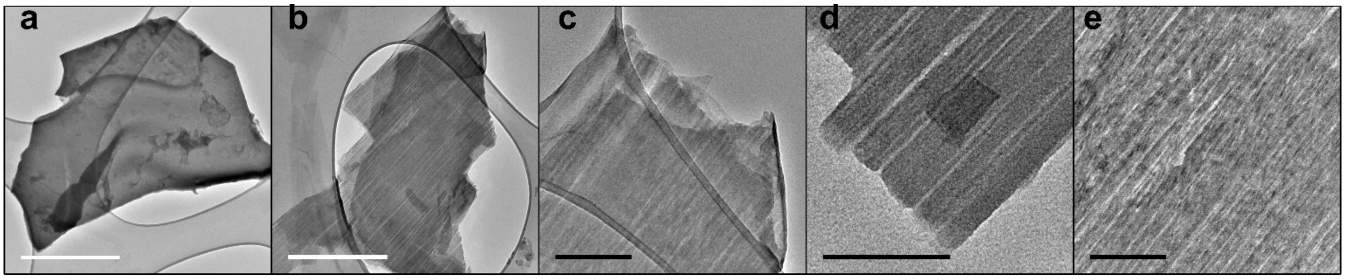


***Figure S10.*** *Additional TEM micrographs of fractured 2D AsPene sheets. Scale bars (a-b) 1 µm (c-e) 200 nm.*

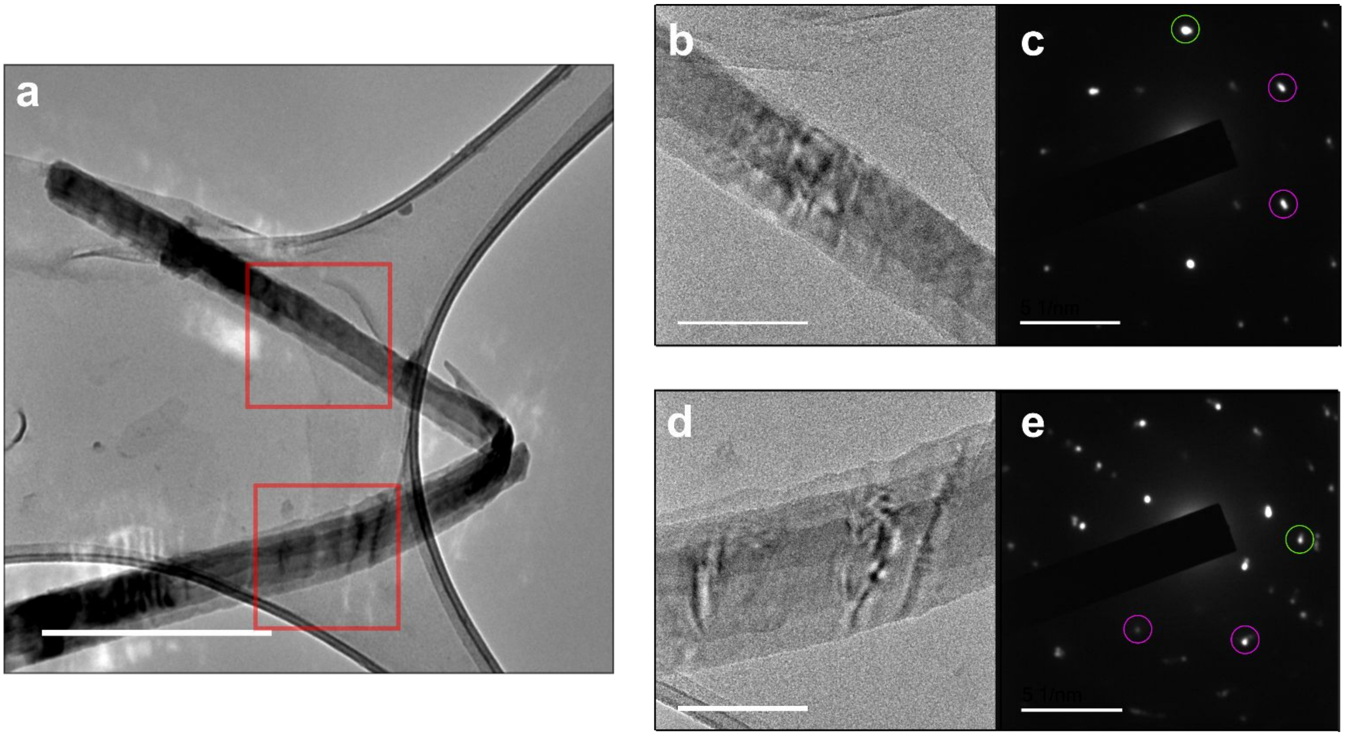


***Figure S11***. *(a) Micrograph of folded few-layered AsPNR, with red squares highlighting magnification areas, scale bar 500 nm (b,d) magnified TEM micrographs, scale bars 100 nm (c,e) SAED of 'b' and 'd' respectively, showing rotation of diffraction peaks concurrent with ribbon rotation, with selected peaks highlighted with coloured circles (green 200; purple 102) as guide to the eye, scale bars 5 nm*$^{-1}$.

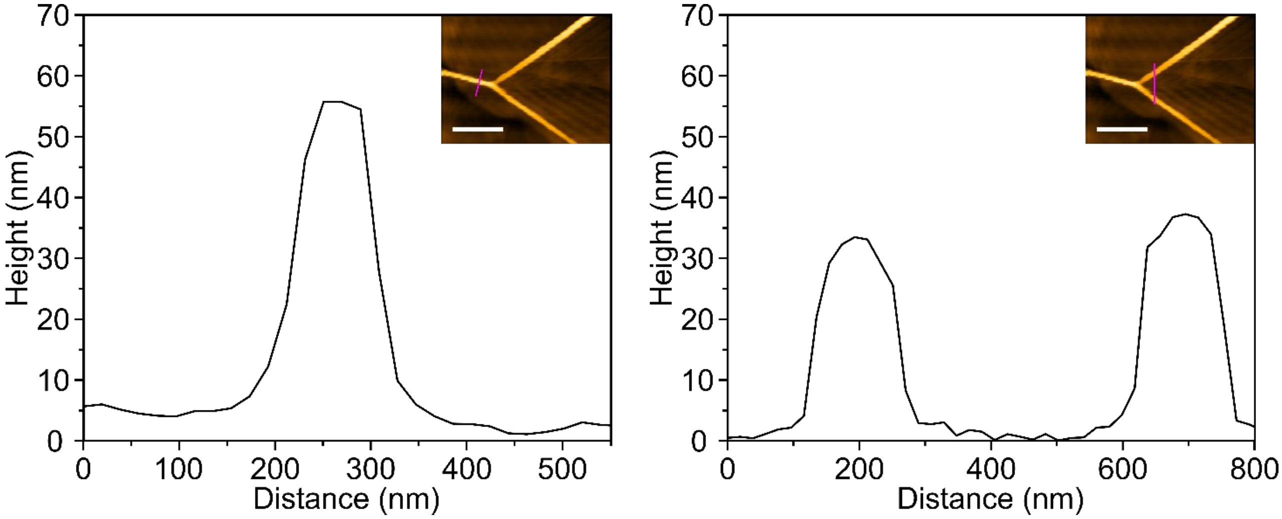


***Figure S12***. *Line cuts of bifurcated AsPNR (main text, Fig. 2p) either side of bifurcation, with AFM micrograph inset with 10 µm xy-scale bar and purple line indicating cut.*

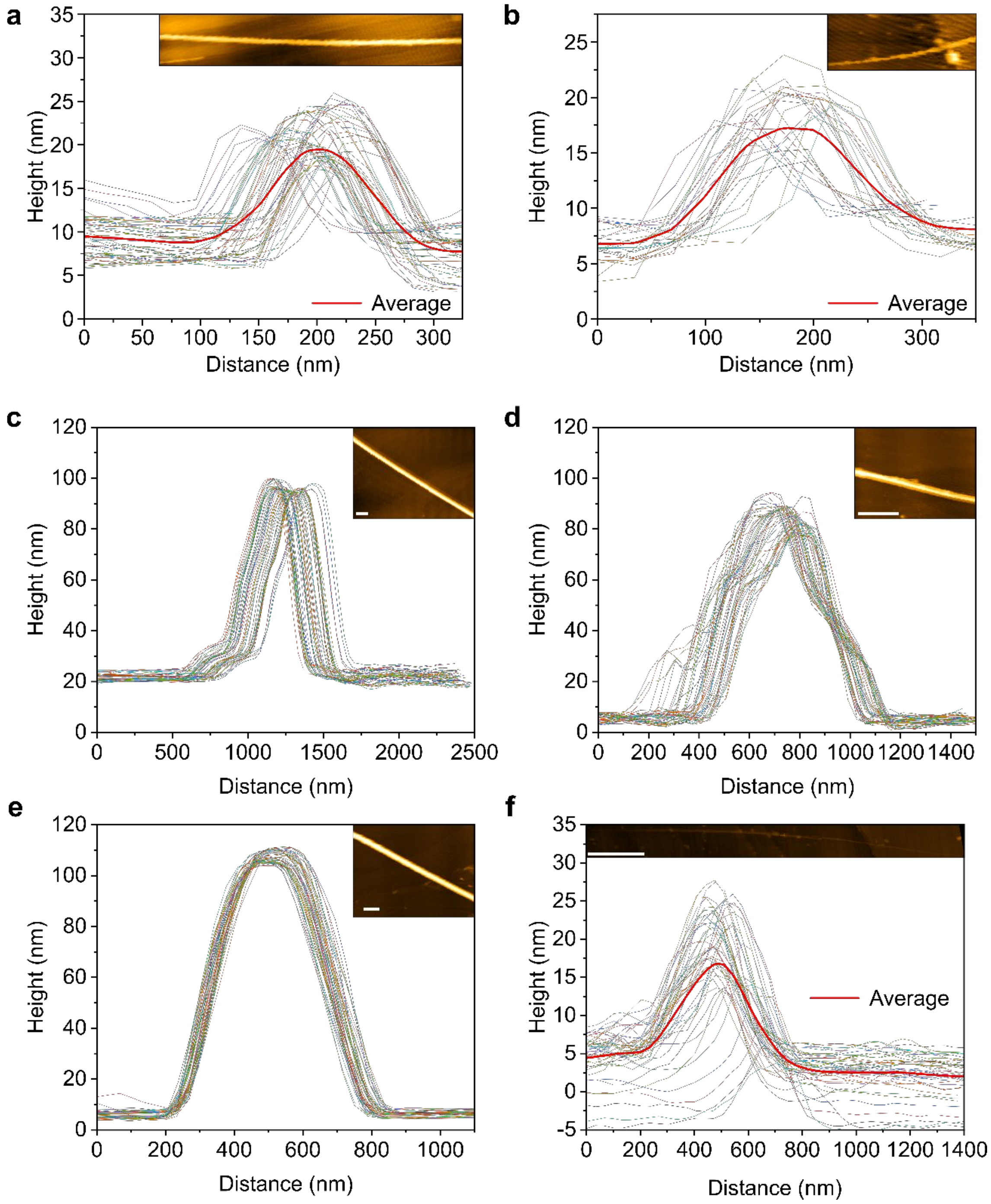


***Figure S13****. Linecuts of AFM micrographs, with micrographs inset with 1 μm scale bars. X line cuts were taken in Y pixels over the length of Z μm of the ribbon: (a) X = 50, Y = 280, Z = 5.22 μm; (b) X = 25, Y = 100, Z = 2.91 μm; (c) X = 50, Y = 241, Z = 8.1 μm; (d) X = 50, Y = 244, Z = 8.2 μm; (e) X = 50, Y = 255, Z = 8.6 μm; (f) X = 45, Y = 415, Z = 109 μm. Due to significant differences in baseline, an averaged linecut is provided for thinner ribbons in a/b/f.*

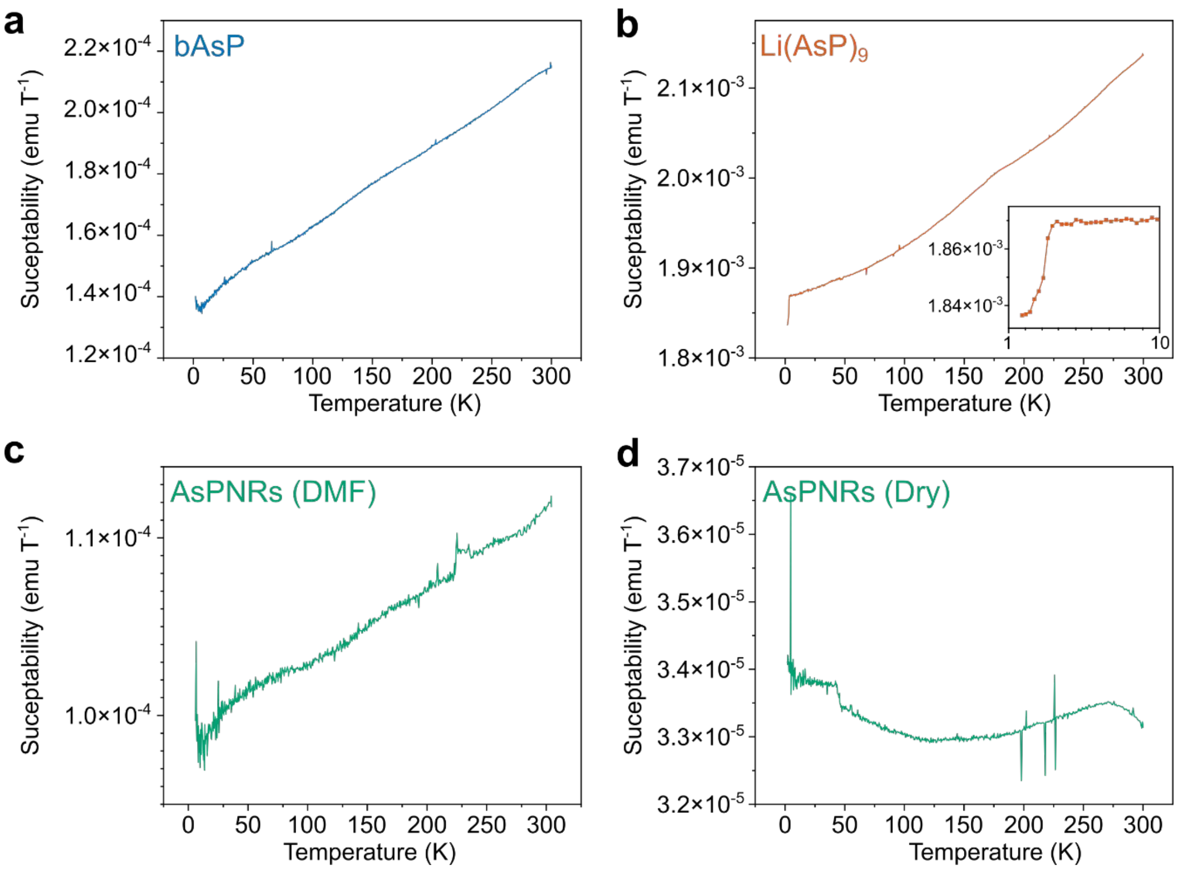


***Figure S14****. Zero field cooled magnetisation measurements in a 50 mT field between 300 – 1.8 K of (a) AsP, (b) Li(AsP)$_9$ with inset highlighting 1 – 10 K region, (c) AsPNRs in DMF solution, (d) AsPNR drop-cast from DMF solution.*

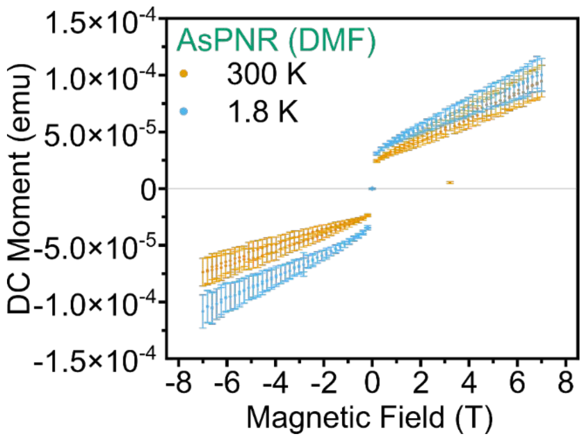


***Figure S15****. Zero field cooled magnetisation measurements in a 50 mT field between 300 – 1.8 K AsPNRs in DMF solution.*

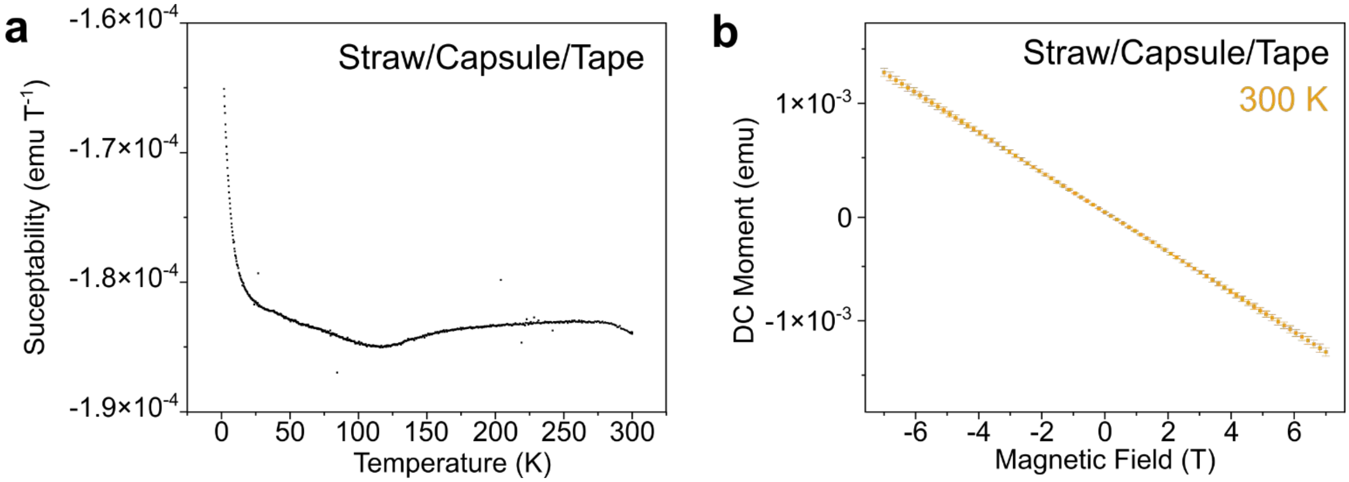


***Figure S16**. Empty holder consisting of a plastic straw, plastic capsule, and Kapton tape. (a) Zero field cooled magnetisation measurements in a 50 mT field at 300 K (b) Isothermal magnetic field sweep of empty control, system showing purely weak diamagnetic behaviour.*

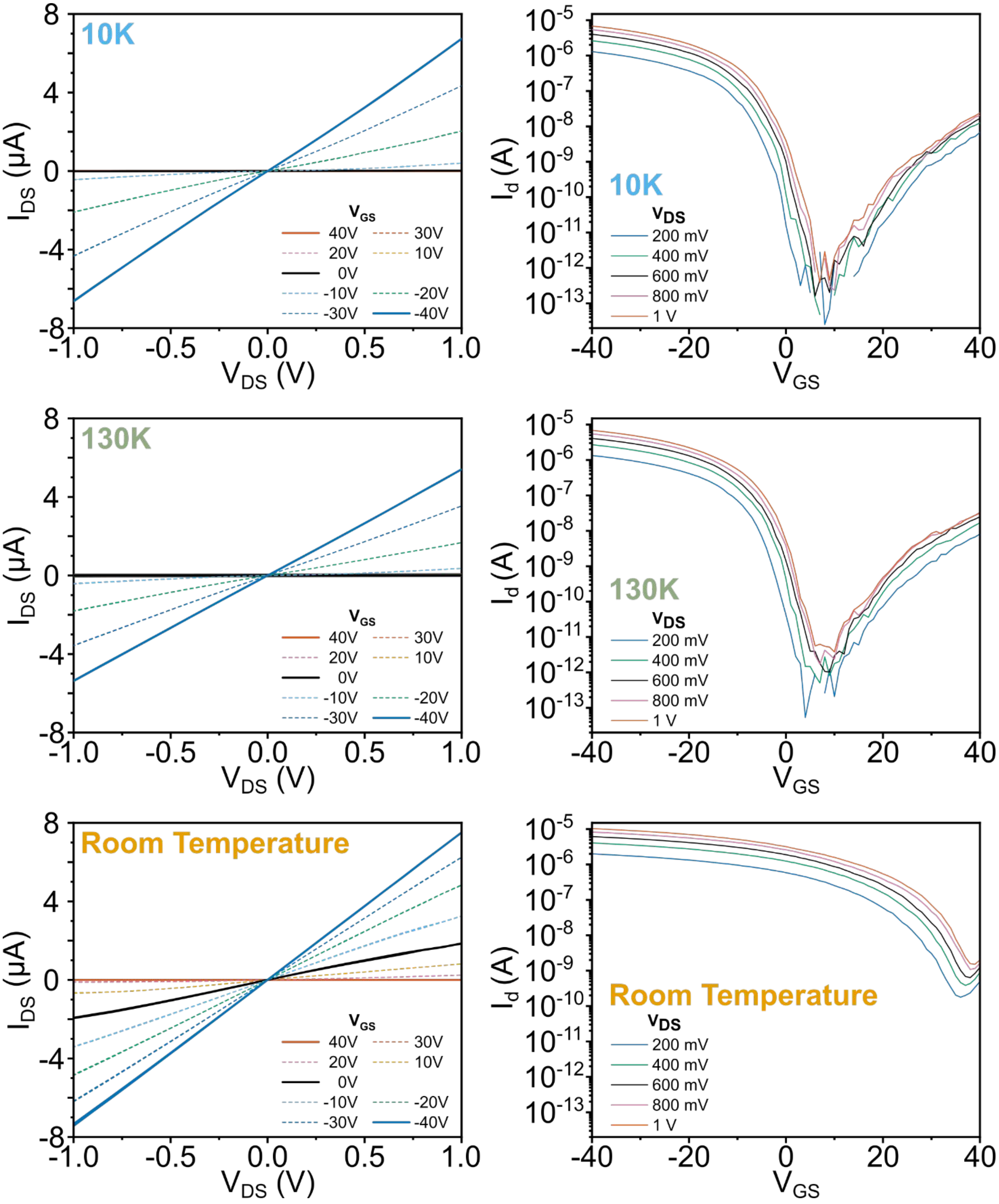


***Figure S17***. *Temperature dependent field-effect mobility at (top) 10K, (middle) 130 K, and (bottom) room temperature.*

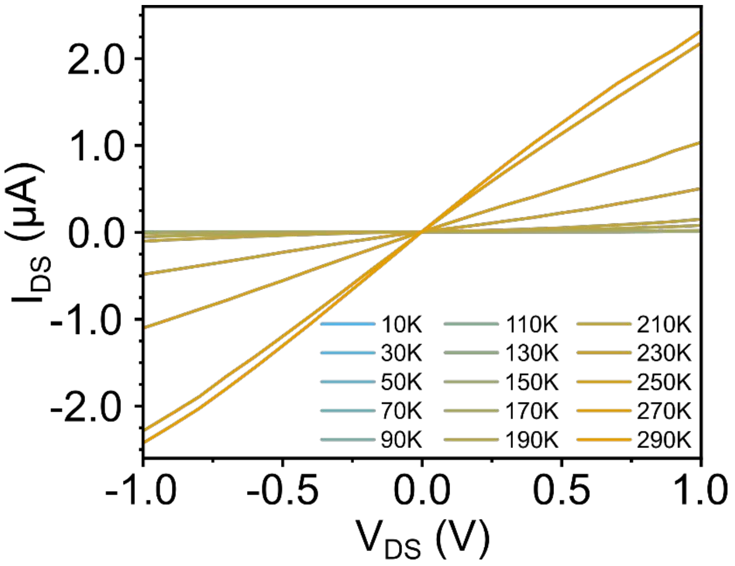


***Figure S18***. *Temperature dependent FET output curves of an AsPNR FET at VGS = 0 V between 10 – 290 K, over full measured voltage range.*

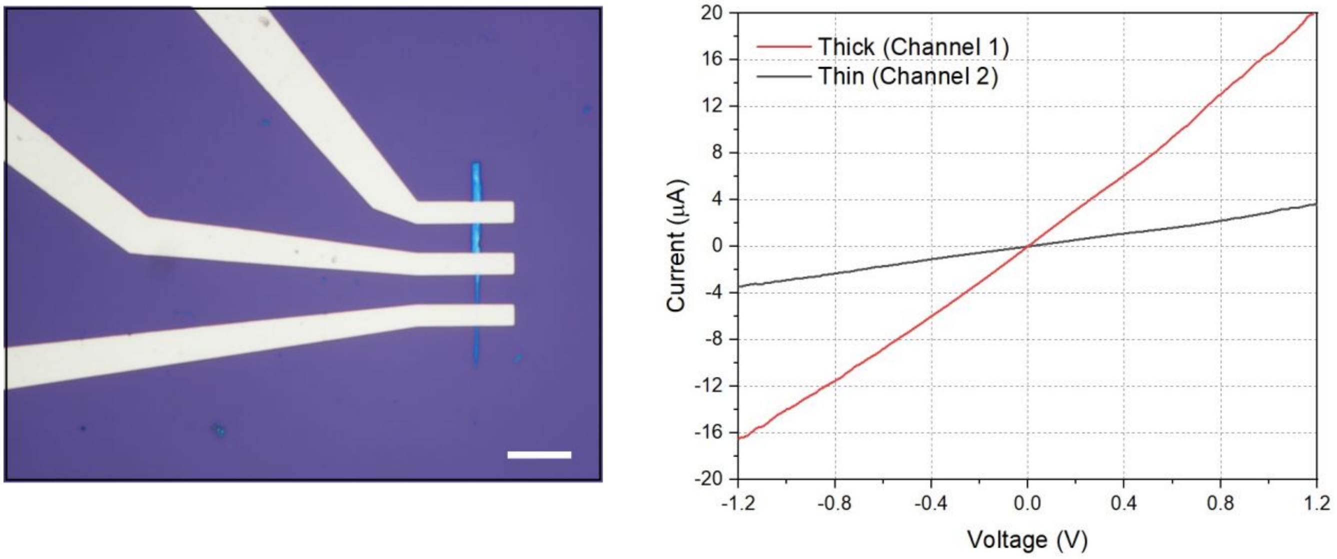


***Figure S19***. *AsPNR FETs. (left) Image, scale bar 10 μm, (right) and temperature dependent FET output curves at $V_{GS}$ = 0 V at 290 K for devices of different widths.*

## Section 2 – DFT Calculations

DFT calculations were undertaken to provide information on the initial bAsP and the intercalated $Li(AsP)_9$, by modelling the parent bP and bAs structures, as well as three configurations of bAsP (x = 0.5 to simplify calculations versus the experimental x = 0.45). The three configurations constituted alternating layers of bAs/bP ("layer"), alternating As/P down each zig-zag chain ("mix"), and a random assignment in a 32 atom cell ("random"). The former two were calculated from starting geometries of the high symmetry bP input, and the latter from a 2 × 2 × 2 supercell of bP. Calculations were undertaken using a B3LYP functional with D3 dispersion correction, owing to previous success in modelling covalent layered crystals. Triple zeta with polarization basis sets were used to provide the best approximation when charged with Li in latter calculations.

As shown in Table S1, the volume (3% change) and energy (<0.03 eV/atom) of the various bAsP models were near consistent, however the structures (Fig. S25) and predicted pXRD (Fig. S22) varied significantly. Due to this variation, the use of theoretical bAsP (and bP/bAs, Fig S21) patterns in comparison to the experimental data only enabled assignment of (020), (021), (040)+(101)+(111), and the twin (131)+(002) peaks (with it unclear which is the lower angle of the two). From the (020), the "random" bAsP model b-axis cell parameter (10.842 Å) is in good agreement with the experimental 5.42 Å interlayer value.

The "random" and "mix" bAsP models showed shearing of the layers to give offset corrugations forming interstitial voids between adjacent layers. This offset configuration was also modelled for bP and bAs ("offset", Fig. S24c-d), and was shown to also be of comparable stability to the original ("overlap") configurations (Table S1). Intercalation of the "random" bAsP with 1/8 eq Li led to stretching in all axes with Li sitting in the inter-corrugation voids, however, the structure changed dramatically between open/closed shell configurations (Fig S26).

***Table S1.*** *Cell properties for DFT modelled systems. From left to right: Cell volume, cell parameters, and formation energy.*

| **Model** | **Volume (Å³)** | **a (Å)** | **b (Å)** | **c (Å)** | **$\Delta H_f$ (eV/atom)** |
|---|---|---|---|---|---|
| bP (overlap) | 73.609 | 3.372 | 10.205 | 4.279 | n/a |
| bAs (overlap) | 87.697 | 3.754 | 10.721 | 4.358 | n/a |
| bP (offset) | 75.45975 | 3.331 | 10.577 | 4.367 | 0.005448 |
| bAs (offset) | 89.41495 | 3.675 | 11.118 | 4.491 | 0.007743 |
| AsP (layered) | 160.532 | 3.562 | 10.307 | 4.373 | 0.028111 |
| AsP (mix) | 159.65 | 3.574 | 10.433 | 4.285 | 0.02491 |
| AsP (random) | 165.13225 | 7.012 | 10.842 | 8.866 | 0.010134 |

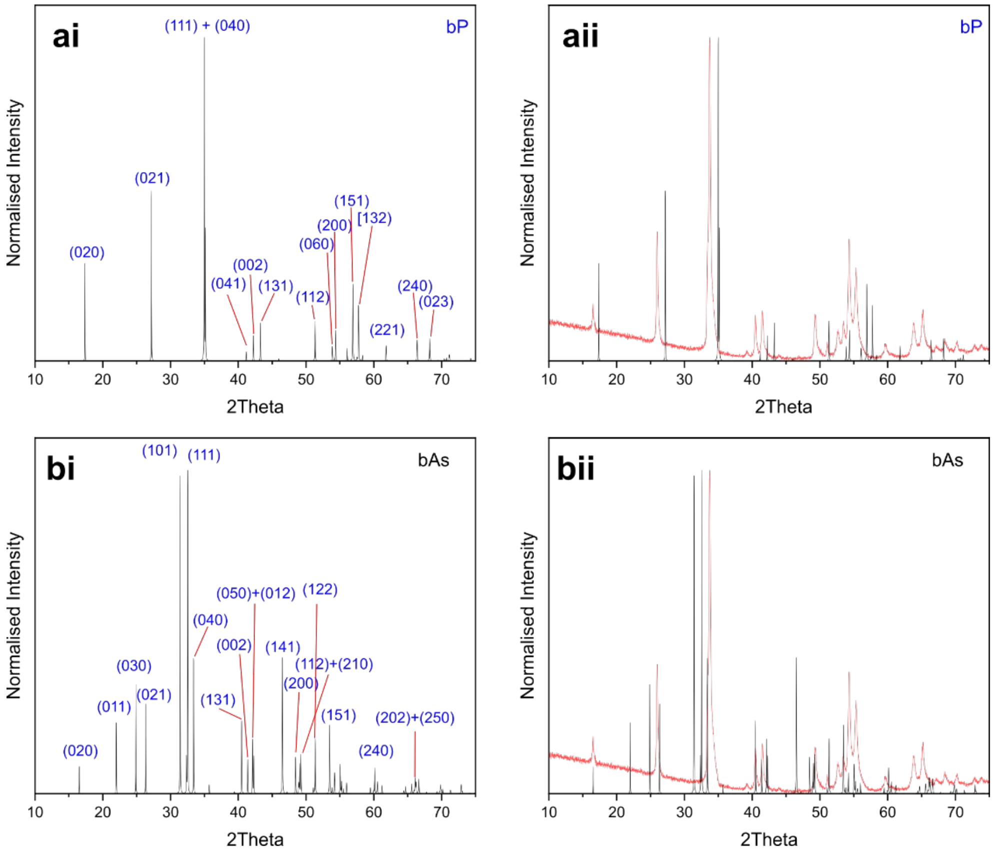


***Figure S21.*** *pXRD patterns predicted from DFT-modelled systems (a) bP, overlapping configuration, (b) bAs, overlapping configuration. Theoretical patterns shown with both (i) indexing, (ii) overlaid experimental data of bAsP.*

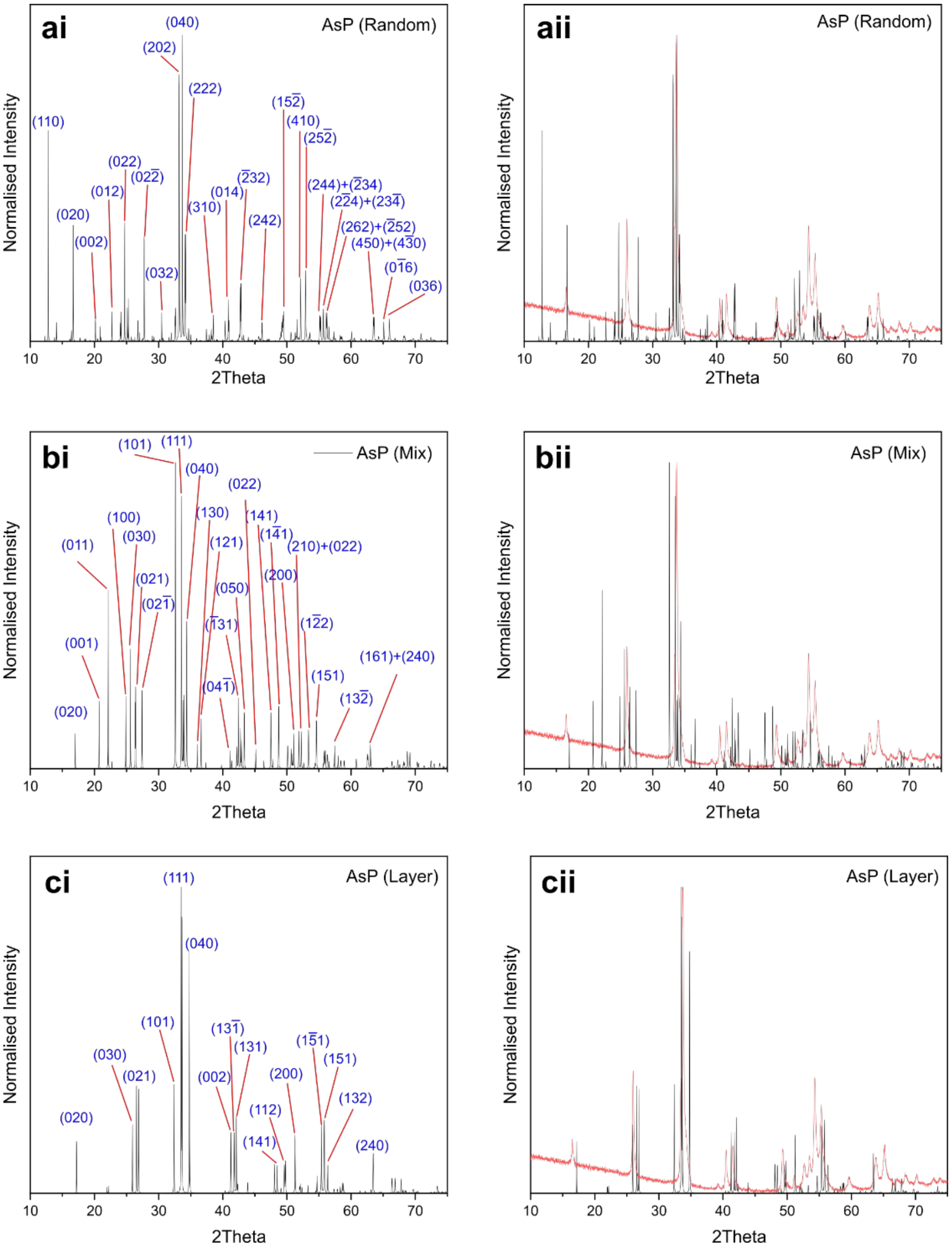


***Figure S22.*** *pXRD patterns predicted from DFT-modelled systems of (a) “Random (b) “Mix” and (c) “Layer” configurations. Theoretical patterns shown with both (i) indexing, (ii) overlaid experimental data of bAsP.*

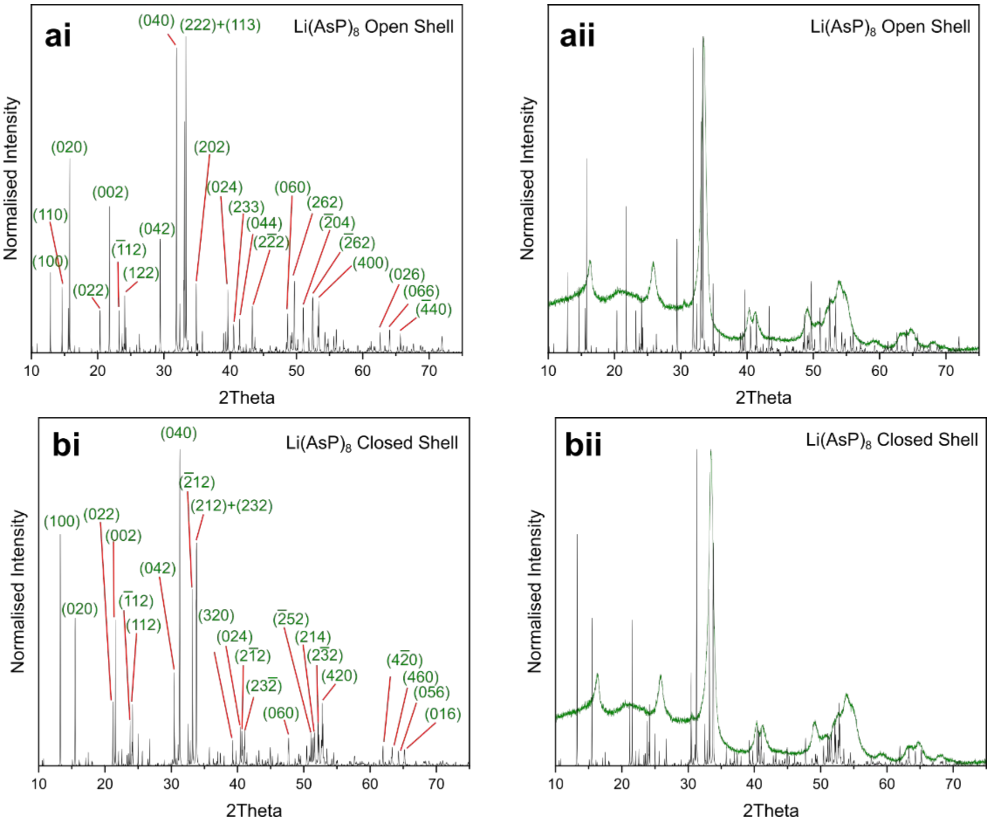


***Figure S23.*** *pXRD patterns predicted from DFT-modelled systems of $Li_4As_{16}P_{16}$ derived from the "Random" bAsP structure, calculated with (a) open shell and (b) closed shell electronic configurations. Theoretical patterns shown with both (i) indexing, (ii) overlaid experimental data of $Li(AsP)_9$.*

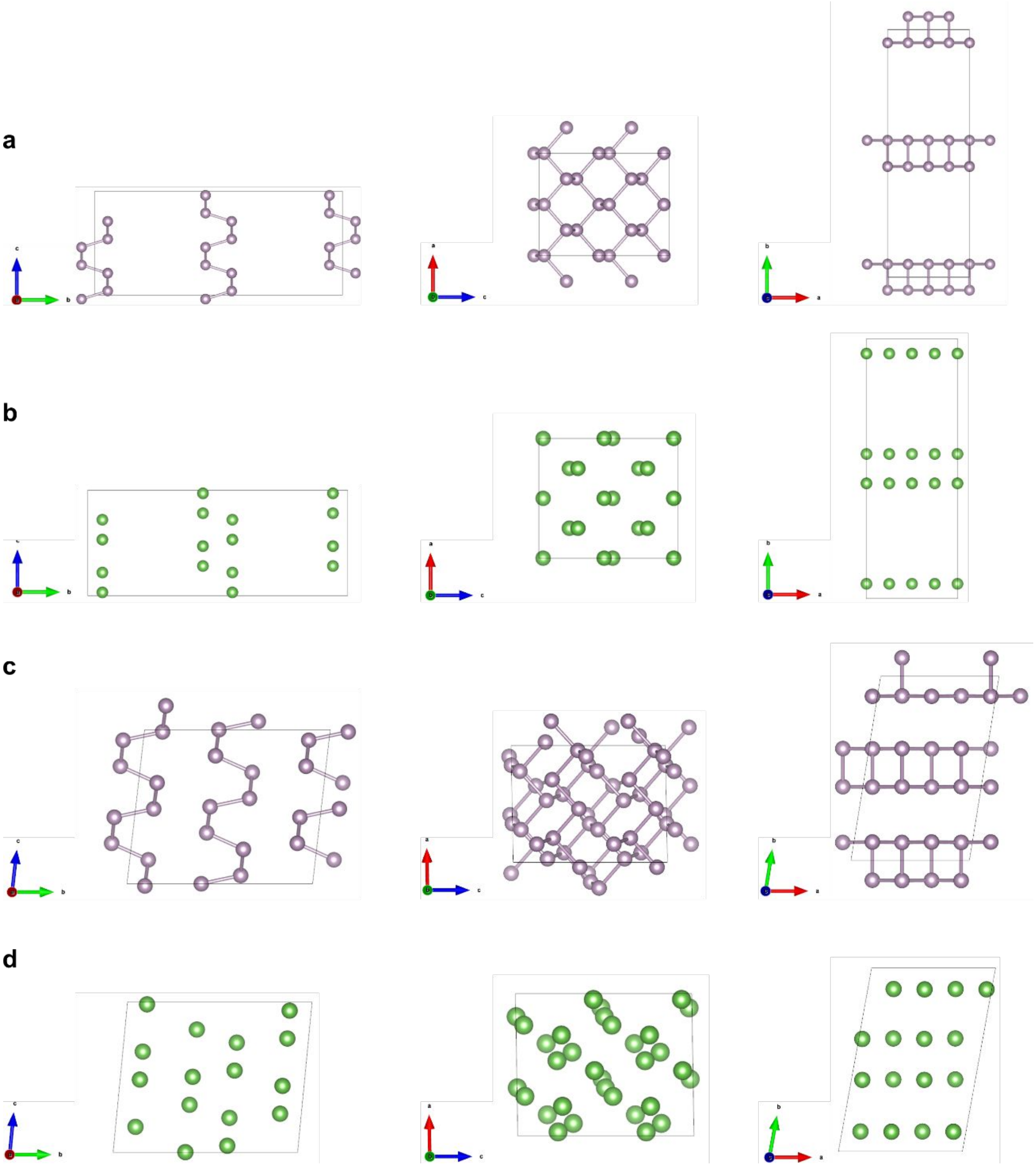


***Figure S24.****Unit cells of pure black P/As modelled from DFT. (a) bP with overlapping layers (b) bAs with overlapping layers. (c) bP with offset layers (d) bAs with offset layers. Shown down (from left to right) a, b, and c-axes*

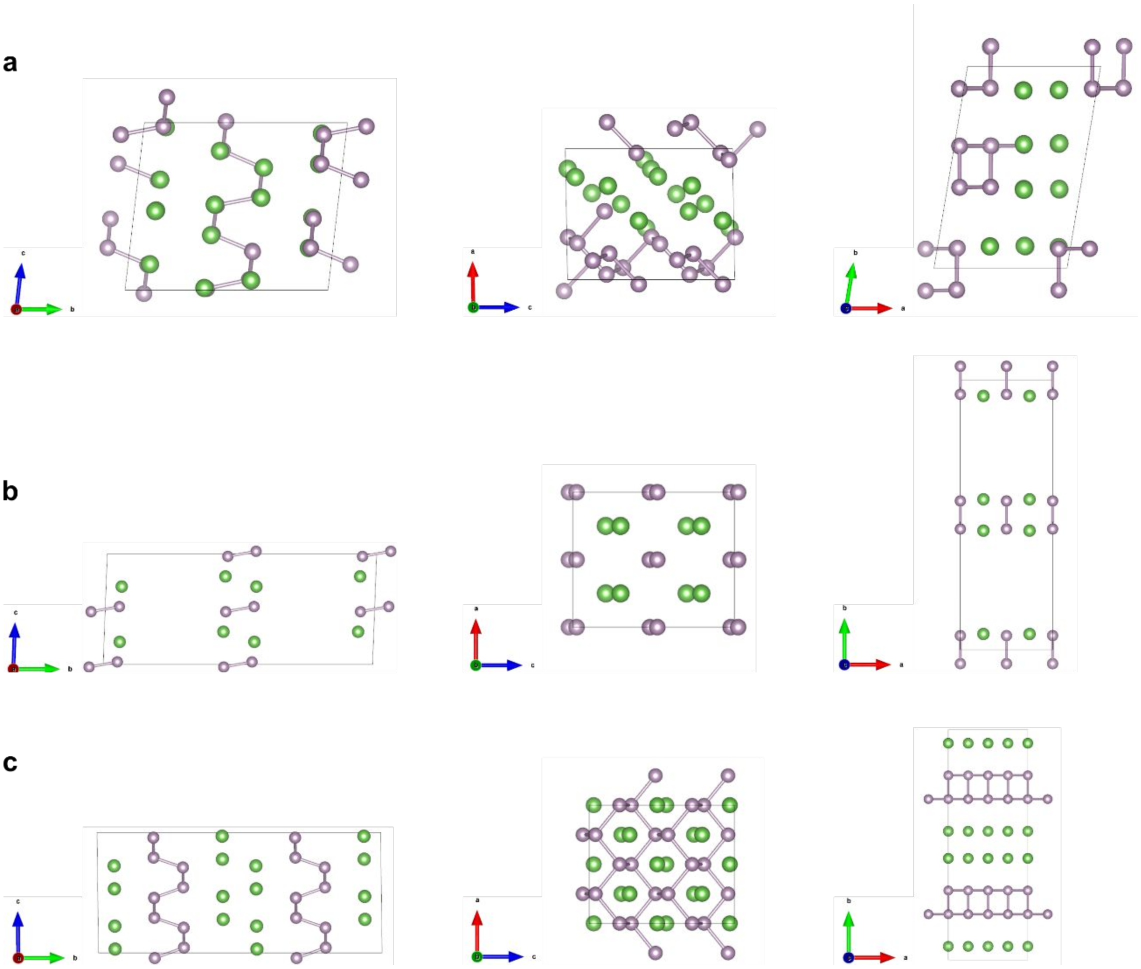


***Figure S25.*** *Unit cells of x=0.5 AsP of different framework arrangements, modelled from DFT. (a) "Random" consisting of randomly assigned As/P in the 32 atom unit cell (b) "Mix" consisting of alternating As/P in the zig-zag chains. (c) "Layer" consisting of alternating pure As and P layers. Shown down (from left to right) a, b, and c-axes*

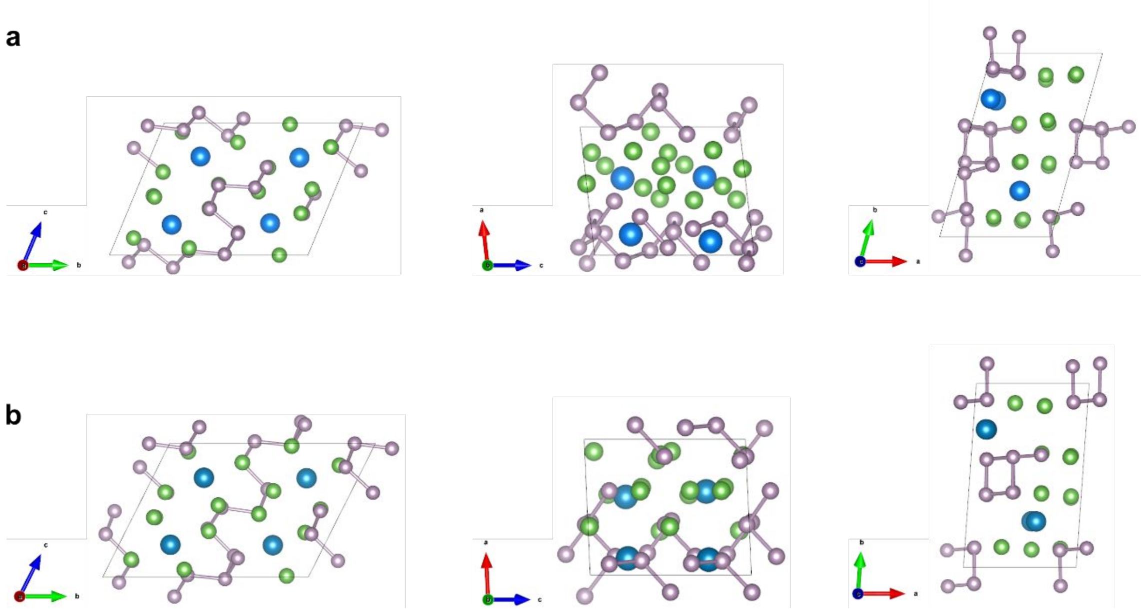


***Figure S26.*** *Unit cells of* $Li_4As_{16}P_{16}$ *modelled from DFT. (a) calculated with closed shell configuration (b) calculated with open shell configuration. Shown down (from left to right) a, b, and c-axes*
